# Distinctive Nuclear Localization Signals in the Oomycete *Phytophthora sojae*

**DOI:** 10.3389/fmicb.2017.00010

**Published:** 2017-02-02

**Authors:** Yufeng Fang, Hyo Sang Jang, Gregory W. Watson, Dulani P. Wellappili, Brett M. Tyler

**Affiliations:** ^1^Interdisciplinary Ph.D. Program in Genetics, Bioinformatics and Computational Biology, Virginia TechBlacksburg, VA, USA; ^2^Center for Genome Research and Biocomputing and Department of Botany and Plant Pathology, Oregon State UniversityCorvallis, OR, USA; ^3^Department of Environmental and Molecular Toxicology, Oregon State UniversityCorvallis, OR, USA; ^4^Molecular and Cellular Biology Program, Oregon State UniversityCorvallis, OR, USA; ^5^Biological and Population Health Sciences, Oregon State UniversityCorvallis, OR, USA

**Keywords:** oomycetes, *Phytophthora sojae*, nuclear localization, nucleus labeling, NLS, classical NLS, PY-NLS

## Abstract

To date, nuclear localization signals (NLSs) that target proteins to nuclei in oomycetes have not been defined, but have been assumed to be the same as in higher eukaryotes. Here, we use the soybean pathogen *Phytophthora sojae* as a model to investigate these sequences in oomycetes. By establishing a reliable *in vivo* NLS assay based on confocal microscopy, we found that many canonical monopartite and bipartite classical NLSs (cNLSs) mediated nuclear import poorly in *P. sojae*. We found that efficient localization of *P. sojae* nuclear proteins by cNLSs requires additional basic amino acids at distal sites or collaboration with other NLSs. We found that several representatives of another well-characterized NLS, proline-tyrosine NLS (PY-NLS) also functioned poorly in *P. sojae*. To characterize PY-NLSs in *P. sojae*, we experimentally defined the residues required by functional PY-NLSs in three *P. sojae* nuclear-localized proteins. These results showed that functional *P. sojae* PY-NLSs include an additional cluster of basic residues for efficient nuclear import. Finally, analysis of several highly conserved *P. sojae* nuclear proteins including ribosomal proteins and core histones revealed that these proteins exhibit a similar but stronger set of sequence requirements for nuclear targeting compared with their orthologs in mammals or yeast.

## Introduction

In eukaryotes, many proteins such as core histones, transcription factors, and ribosomal proteins must be transported into the nucleus to accomplish their functions. Transport of those proteins into the nucleus occurs through large, proteinaceous structures called nuclear pore complexes (NPCs). Generally, NPCs allow passive diffusion of molecules smaller than 40-60 kDa, but require an appropriate sorting signal for passage of larger proteins (Lange et al., 2007). The sorting signals carried by those proteins are called nuclear localization signals (NLSs), that generally are short stretches of amino acids recognized by nucleo-cytoplasmic transporters (karyopherins) that promote active transport of proteins into the nucleus (Xu et al., 2010; Marfori et al., 2011). To date, two principal types of NLSs have been defined: the classical NLS (cNLS) and the proline-tyrosine NLS (PY-NLS).

cNLSs are the best-characterized nuclear targeting signals, and are recognized by karyopherin-β1 (Importin-β, Kapβ1) through direct binding to an adaptor protein karyopherin-α (Importin-α, Kapα) (Lange et al., 2007; Marfori et al., 2011). According to the numbers of basic amino acid clusters within them, cNLSs are further divided into two subclasses, monopartite, and bipartite cNLSs. Monopartite cNLSs contain a single stretch of basic amino acids which may consist of at least four consecutive basic amino acids, exemplified by the SV40 large T antigen NLS (PKKKRKV) (Kalderon et al., 1983, 1984). Alternatively, three non-consecutive basic amino acids may suffice, exemplified by the *c-Myc* proto-oncoprotein NLS (PAAKRVKLD) (Makkerh et al., 1996). Traditionally, the monopartite cNLS has a consensus of K(K/R)X(K/R) (Lange et al., 2007). Bipartite cNLSs have two stretches of basic amino acids separated by 10–12 amino acids (Lange et al., 2007). They were first found in *Xenopus* nucleoplasmin (KRPAATKKAGQAKKKK) (Dingwall et al., 1982) and are represented by the consensus sequence (K/R)(K/R)X_10−12_(K/R)_3/5_ (X is any amino acid and (K/R)_3/5_ represents three lysine or arginine residues out of five consecutive amino acids) (Dingwall and Laskey, 1991). With increasing numbers of cNLS-bearing proteins identified, and more in-depth biochemical and biophysical analyses, the cNLS consensus sequences have been progressively expanded (reviewed in Marfori et al., 2011).

The PY-NLS is recognized for nuclear import by karyopherin-β2 (Kapβ2) in humans and by its ortholog Kap104 in yeast (Lee et al., 2006). Compared to the cNLS, fewer PY-NLS proteins have been characterized experimentally (~42 through 2015, Soniat and Chook, 2015). PY-NLSs are generally longer (15–30 residues) and more variable than cNLSs, making it more difficult to clearly define their common features (Xu et al., 2010; Chook and Suel, 2011). M9NLS is the best-characterized PY-NLS. It consists of a 38-residue domain from a splicing factor, heterogeneous nuclear ribonucleoprotein A1 (hnRNP A1) (Bonifaci et al., 1997; Truant et al., 1999). On the basis of the crystal structure of human Kapβ2 bound to M9NLS, Lee et al. (2006) proposed three rules for PY-NLSs: (1) structurally disordered in free substrates; (2) overall basic character; and (3) central hydrophobic or basic motif (epitope 1) followed by the motif R/H/K-X_2−5_-PY (the R/H/K- and PY-motifs are defined as epitopes 2 and 3 respectively). According to the composition of the amino acids in epitope 1, PY-NLSs have been further classified into hydrophobic and basic PY-NLSs (hPY- and bPY-NLSs) (Lee et al., 2006). Later, the rules were updated to accommodate additional features identified in *Saccharomyces cerevisiae*: the Kapβ2 ortholog Kap104 only recognizes the basic but not the hydrophobic PY-NLS (Süel et al., 2008); and the tyrosine in the C-terminal PY epitope (epitope 3) displays degeneracy; not only PY but also some other motifs like PL could be recognized by yeast Kap104 (Süel et al., 2008). *In silico* predictions suggest that Kapβ2-mediated import accounts for a substantial fraction of substrates involved in RNA processing and transcription factors (Süel and Chook, 2009; Chook and Suel, 2011).

Based on a growing volume of functional data from human and yeast nuclear translocation signals, a number of prediction software packages have been developed to bioinformatically predict nuclear-localized proteins and the responsible signals. Those tools can be classified into two general groups, namely classification of subcellular or nuclear localization; and detection of NLS sequences (Marfori et al., 2011). Software such as *WoLF PSORT* (Horton et al., 2007) and *NucPred* can be used for predicting if proteins of interest have nuclear localization based on the general properties of the proteins, while *PSORT II* (PSORT, 1997), *NLStradamus* (Nguyen Ba et al., 2009), and *cNLS Mapper* (Kosugi et al., 2009) predict residues that may serve as NLSs. *PredictNLS* (Cokol et al., 2000) provides prediction for both subcellular localization and the NLS sequences of given proteins. Of those NLS predictors, *PSORT II* and its extension *WoLF PSORT* are the two most widely adopted tools that are built on NLS consensus sequences (PSORT, 1997; Horton et al., 2007). In contrast, *NLStradamus* is based on experimentally validated NLSs in yeast (Nguyen Ba et al., 2009), and *cNLS Mapper* is based on synthetic peptide library data (Kosugi et al., 2009). Most of the computational tools to date are designed for prediction of cNLS, except *NLStradamus*, which does not distinguish the NLS types.

*Phytophthora sojae* is a destructive oomycete pathogen that infects soybean seedlings as well as established plants (Tyler, 2007). Although oomycetes physiologically and morphologically resemble fungi, molecular taxonomy has shown that oomycetes are phylogenetically close to algae and diatoms (Tyler, 2007; Kamoun et al., 2015). Oomycetes are diploid and lack a free haploid life stage. The genomes of oomycetes (50–250 Mb) are also generally larger than those of true fungi (10–40 Mb) (reviewed in Judelson and Blanco, 2005). Most *Phytophthora* species are plant pathogens and together damage a huge range of agriculturally and ornamentally important plants (Erwin and Ribeiro, 1996). For instance, *P. infestans*, causes the potato late-blight disease that resulted in the Irish potato famine, and continues to be a problem for potato and tomato crops (Judelson and Blanco, 2005). *P. sojae* causes around $1-2 billion in losses per year to the soybean crop (Tyler, 2007). Because of its economic impact, *P. sojae*, along with *P. infestans*, has been developed as a model species for the study of oomycete plant pathogens (Tyler, 2007).

To date, most nuclear localization studies have been carried out in model organisms, and no NLS sequences have yet been defined in oomycetes. Here we find that many eukaryotic NLS sequences function poorly if at all in *P. sojae*. We demonstrate that efficient localization of *P. sojae* nuclear protein by cNLSs requires additional basic amino acids at distal sites or collaboration with other NLSs. Furthermore, we show that a fully functional PY-NLS requires additional basic residues either within the motif itself or adjacent to the motif. Finally, comparison of the nuclear localization activities of NLS sequences from *P. sojae* ribosomal proteins and core histones with those from other eukaryotes reveals that *P. sojae* may use modified nuclear import mechanisms for those highly conserved nuclear proteins.

## Materials and methods

### *P. sojae* strains and growth conditions

All of the NLS tests were carried out in the *P. sojae* reference isolate P6497 (race 2). Cultures were routinely grown and maintained in cleared V8 medium at 25°C in the dark. *P. sojae* transformants were incubated in 12-well plates containing liquid V8 media supplemented with 50 μg/ml G418 (Geneticin) for 2–3 days before examination by confocal microscopy.

### Sequence analysis

*Phytophthora* protein sequences and IDs were obtained from FungiDB (Stajich et al., 2012). Their corresponding accession numbers in the GenBank database are listed in Table S1. Ribosomal protein and core histone sequences of *Arabidopsis*, yeast and human were obtained from the GenBank database: *Arabidopsis* L27a (accession number, NP_177217), H3.1 (NP_201339), H4, (NP_190179); human H3.k (P68431), H4.j (AAA52652), L27a (NP_000981); *Saccharomyces cerevisiae* L28 (NP_011412), H3 (or Hht2p, CAY82162); H4 (or Hhf2p, NP_014368). Protein sequence alignments were carried out using *Clustal Omega* (Sievers et al., 2011). Additional sequence information for the proteins PHYSO_357835, PHYSO_480605, PHYSO_251824, PHYSO_561151, and PHYSO_533817 can be found in the Data Sheet 1 (Supplemental Sequences).

### Construction of plasmids

All the primers used in this study are listed in Table S2 in the Supplementary Material. All the fusion protein reporter constructs in which the NLS was fused to the N-terminus of GFP or 2XGFP were based on the plasmid backbone pYF2-2XGFP (Fang and Tyler, 2016). To test reporters with the NLS fused to the C-terminus of GFP, a new plasmid backbone pYF3-2XGFP was generated by inserting an *eGFP* fragment with attached C-terminal multiple cloning sites and stop codon (*Bsr* GI-*Hpa* I-*Bsp* EI-*Mlu* I-TAA) into the *Afl* II and *Apa* I sites of the backbone pYF2-GFP. To clone NLS candidates efficiently and economically, NLSs derived from exogenous genes smaller than ~80 bp were created by oligo annealing and inserted into the *Spe* I and *Sac* II sites of pYF2-2XGFP, or the *Bsr* GI and *Bsp* EI sites of pYF3-2XGFP. PCR products from *P. sojae* genes were inserted into the *Stu* I site of pYF2-2XGFP or the *Hpa* I site of pYF3-2XGFP by blunt ligation. The *P. sojae* H2B nuclear marker fusions (H2B-GFP and H2B-mCherry) and the *P. capsici* fibrillarin nucleolar marker fusion (FIB-mCherry) were created using the pGFPN or pMCherryN plasmids (Ah-Fong and Judelson, 2011). Point mutations and deletions of DNA sequences were made through QuikChange Lightning Multi Site-Directed Mutagenesis (Agilent Technologies).

To express 2XGFP fusions in mammalian cells, genes encoding 2XGFP and its SV40 NLS and M9NLS fusions were PCR-amplified from *P. sojae* expression plasmids using primers pYF2_GW_F and GFP_stop_GW_R, and integrated into pcDNA 3.2/V5-DEST by Gateway™ cloning (Thermo Scientific). Integration of 2XGFP into pcDNA 3.2/V5-DEST also generated a plasmid backbone pcDNA 3.2-2XGFP, in which a preserved *Eco* RV site originating from the pYF2 backbone was used for insertion of PY-NLS amplicons by blunt ligation.

PCR-amplification was conducted using Phusion® High-Fidelity DNA Polymerase (NEB). Standard molecular techniques were performed as described by Sambrook and Russell (2001) or according to instructions from kit manufacturers.

### *P. sojae* transformation

Polyethylene glycol (PEG)-mediated protoplast transformations were conducted by an improved protocol as described previously (Fang and Tyler, 2016). In general, *P. sojae* protoplasts were isolated by enzyme digestion using 0.5% Lysing Enzymes from *Trichoderma harzianum* (Sigma L1412) and 0.5% CELLULYSIN® Cellulase (Calbiochem 219466) in 0.4 M mannitol, 20 mM KCl, 20 mM MES, pH 5.7, 10 mM CaCl_2_. One milliliter of protoplasts at a density of 2 × 10^6^–2 × 10^7^/ml and 20–30 μg DNA were used for single plasmid transformations. For co-transformation experiments, 20–30 μg of the plasmid carrying the *NPT II* selectable marker gene was used, together with an equimolar ratio of any other DNAs included. Protoplasts were regenerated overnight in regeneration media (pea broth containing 0.5 M mannitol). Regenerated protoplasts were collected and grown as colonies in solid regeneration media containing 50 μg/ml G418 (Geneticin) for 2 days. G418-resistant colonies were transferred to 12-well plates containing liquid V8 media supplemented with 50 μg/ml G418 and incubated for 2–3 days at 25°C before confocal microscopy observation. Under these conditions, *P. sojae* transformant colonies typically continue to express genes introduced on exogenous DNA for 1–3 weeks, even if the genes are not integrated into the chromosomes. Here we did not test whether the exogenous DNA had integrated in the chromosomes; thus we refer to the mycelial colonies recovered by our procedure as transient transformants.

### Human cell line transfection and immunocytochemistry

HEK293 cells were maintained in DMEM media (Mediatech) supplemented with 10% fetal bovine serum (Tissue Culture Biologicals), penicillin-streptomycin solution (1 IU/ml penicillin and 100 ug/ml streptomycin, Mediatech). Transfection was done according to the manufacturer's protocol. Briefly, HEK293 cells were plated on a glass coverslip in a 24-well plate (Greiner Bio-One) at a density sufficient for 70% confluency at the time of transfection. Cells were transfected with 500 ng DNA and 1 μl of Lipofectamine 2000 (Invitrogen) per well for 48 h. For immunocytochemistry, the transfected cells were fixed with 3.7% (w/v) paraformaldehyde in PBS for 15 min at RT. The fixed cells were permeabilized with 0.1% Triton X-100 in PBS for 10 min at RT and incubated with 1% bovine serum albumin in PBS for 1 h at RT. Then, the cells were incubated with rabbit anti-GFP antibody conjugated with AlexaFluor 488 (Invitrogen) overnight at 4°C. Cells on the coverslip were mounted on a glass slide using ProLong Gold antifade with DAPI (Invitrogen) and dried overnight at RT in darkness.

### Confocal microscopy imaging

Laser scanning confocal microscopy (Zeiss LSM 780 NLO) was used to monitor the subcellular localization of fluorescent protein fusions in *P. sojae* and human cell transformants. Clumps of 2–3 days old transformed *P. sojae* mycelia grown in liquid V8 media were removed with a toothpick, washed, and maintained in modified Plich media (0.5 g KH_2_PO_4_, 0.25 g MgSO_4_•7H_2_O, 1 g asparagine, 1 mg thiamine, 0.5 g yeast extract, 10 mg β–sitosterol, 25 g glucose dissolved in 1 l water) before observation. For DAPI staining, mycelia samples were pre-treated with Phosphate-buffered saline (PBS, pH 7.4) containing 0.2 μg/ml DAPI for 25 min in dark, and washed twice with PBS according to Hardham (2001). Images were captured using a 63X oil objective with excitation/emission settings (in nm) of 405/410–490 for DAPI, 488/504–550 for GFP, and 561/605–650 for mCherry. For each sample, at least three independent *P. sojae* transformants were examined. Images were adjusted using the microscope's built-in Zen 2012 software (Blue and/or Black edition according to different purposes). Images were cropped, and the tonal range was increased by adjusting highlights and shadows without altering the color balance.

To quantitate the ratio of nuclear to cytoplasmic localization, nuclear and cytoplasmic intensities were collected by manually defining the nuclear and cytoplasmic regions in each image as described in Hunter et al. (2014). The mean intensity values of the defined regions were generated by the Zen 2012 (Blue edition) “measure” tool, which ignores any adjustments of tonal range. In most cases, nuclei were identified by the morphology of the GFP-stained region (a large, uniformly stained, irregular ovoid region, often with an unstained nucleolus), and were not confirmed by co-expression of H2B-mCherry. Where nuclei could not be identified due to very poor nuclear localization, H2B-mCherry was co-expressed to verify the nuclei. The nuclear to cytoplasmic fluorescence ratio was calculated using the means (± standard error) of the log_2_ transformed ratios from 30 pairs of adjacent nuclear and cytoplasmic regions, chosen at random from each of three independent *P. sojae* transformants. This procedure produced the log_2_ (nuclear to cytoplasmic ratio) or LNC used to characterize the nuclear localization activity of most constructs in this study. Significance was tested with two-sample, unpaired *t*-tests of the LNC-values performed by the software GraphPad Prism 7, and a false discovery rate (FDR)-adjusted *p*′-value (Benjamini and Hochberg, 1995) of 0.001–0.05, as noted in each figure legend.

## Results

### Establishment of reliable fluorescent labeling of *P. sojae* nuclei for assay of nuclear localization

To assess the activity of NLSs, we implemented a classic *in vivo* NLS assay, in which a candidate NLS was fused to GFP or to two fused GFP moieties (2XGFP) and then transiently expressed in individual *P. sojae* transformants. The fusion of two GFP molecules created a protein (~55 kDa) larger than the NPC threshold for passive diffusion. Subcellular localization of the proteins was visualized by live-cell imaging in at least three individual transformant colonies per construct, using confocal laser scanning microscopy. To assist in verifying the nuclear localization of a protein, the commonly used nuclear dye DAPI (4′, 6-diamidino-2-phenylindole) was initially employed, because it had been reported to label the nuclei of living Hardham, 2001; Zhang et al., 2012 or fixed (Gamboa-Mélendez et al., 2013) *Phytophthora* tissues. However, when living *P. sojae* hyphae were stained with DAPI, the NLS-fused GFPs were extensively distributed into the cytoplasm (Figure S1A). This contrasted with the clear nuclear localization observed in hyphae that were not stained with DAPI (Figure S1A). In particular, when using a 2XGFP reporter, fused to a strong synthetic NLS, PsNLS (Fang and Tyler, 2016), we noticed that regions of hyphae with poor DAPI staining exhibited strong GFP nuclear localization while regions of hyphae well-stained with DAPI showed poor GFP nuclear localization (Figure S1B). This suggested that DAPI staining caused mis-localization of nuclear-localized proteins to the cytoplasm. Indeed, a time-lapse experiment tracking a hypha during DAPI staining showed that after 18 min staining, the nuclei disintegrated and the nuclear-localized PsNLS-2XGFP was released into the cytoplasm (Figure S1C and Supplemental Video 1).

**Figure 1:**
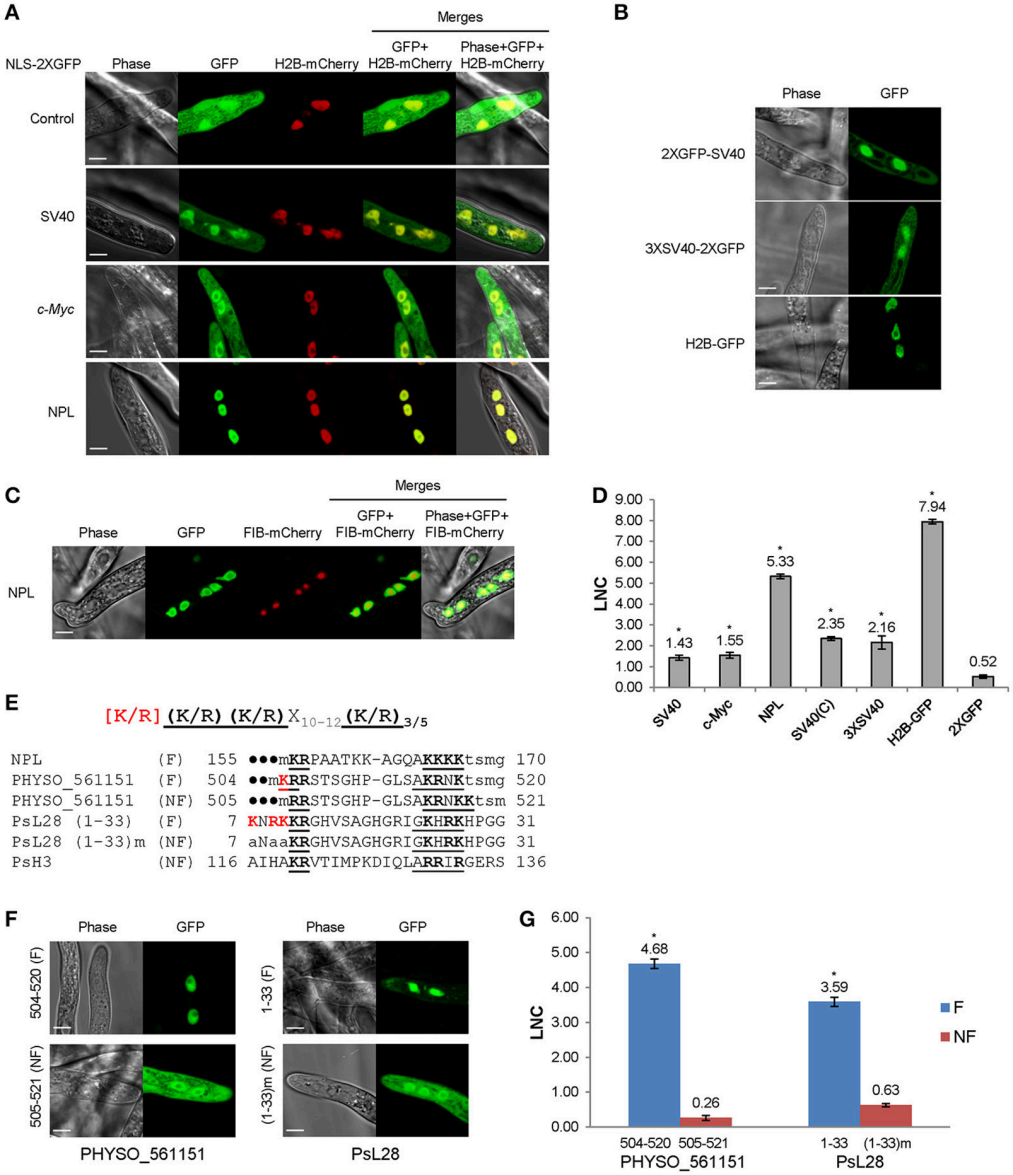
**Functional characterization of monopartite and bipartite cNLSs in *P. sojae* transformants. (A)** Subcellular localization of N-terminal cNLS-2XGFP fusions, involving mammalian cNLS sequences. SV40, *c-Myc*, NPL, represent cNLSs identified in SV40 large T antigen, human *c-Myc* proto-oncoprotein, and *Xenopus* nucleoplasmin respectively; H2B-mCherry = *P. sojae* histone H2B fused to the N-terminus of mCherry; Control = 2XGFP. “GFP” above the panel indicates visualization of the relevant GFP or 2xGFP fusions. Scale bar corresponds to 5 μm in this and all subsequent figures. **(B)** Subcellular localization of different fusions with the SV40 NLS. H2B-GFP was used as a positive control. **(C)** Exclusion of NPL-2XGFP from the nucleolus, as confirmed by *P. capsici* fibrillarin fused to mCherry (FIB-mCherry). **(D)** Quantification of nuclear localization by protein segments shown in **(A,B)**. For quantification in all figures, LNC indicates the mean log_2_-transformed nuclear fluorescence to cytoplasmic fluorescence ratio from ~30 randomly selected pairs of nuclear and adjacent cytoplasmic regions. SV40 (C) = SV40 NLS attached at the C-terminus of 2XGFP (i.e., 2XGFP-SV40 NLS). Error bars, S.E. Asterisks, LNC-values of NLS-GFP fusions that are significantly greater than the LNC of 2XGFP alone (FDR *p*′ < 0.001). **(E)** Sequence alignment of predicted bipartite cNLSs tested in *P. sojae*. The proposed *P. sojae* bipartite cNLS consensus is shown on the top; [K/R] in red indicates the position of additional positive residues required in *P. sojae*. In the sequences of each protein, the two elements of each bipartite cNLS predicted by *PSORT II* (PSORT, 1997) are underlined, and basic amino acids within each cluster are in bold. The additional basic amino acids demonstrated to contribute to nuclear localization are in bold red. The residues in lowercase indicate non-native residues flanking the candidate NLS in each construct. F, functional NLS; NF, non-functional NLS. For PHYSO_561151 and PsL28, both the full length functional cNLSs and the truncated or mutant non-functional cNLSs are shown. **(F,G)** Functional tests of *P. sojae* bipartite NLSs listed in **E**. **F**, representative images. **G**, quantification. (1–33) m indicates mutation of the red-highlighted residues in PsL28 (K7A/R9A/K10A). Representative images are shown in **(A–C,F)**.

To identify a reliable strategy to label *P. sojae* nuclei, we tested other nuclear staining dyes, such as Hoechst 33342. However, *P. sojae* hyphae were not permeable to that dye (data not shown). We also tried to stain the nuclei of fixed hyphae using DAPI according to a protocol described by Gamboa-Mélendez et al. (2013), but redistribution of nuclear GFP into the cytoplasm was still commonly observed. Therefore, we sought to label *P. sojae* nuclei by expressing a nuclear-targeted fluorescent protein. *P. sojae* core histone H2B fused to mCherry was selected according to Ah-Fong and Judelson (2011), and this fusion showed predominant nuclear localization in *P. sojae* hyphae (Figure 1A). Expression of H2B-mCherry appeared to cause some toxicity to *P. sojae*, because reduced numbers of transiently transformed *P. sojae* colonies were obtained with this construct. Similar results were observed with a *P. sojae* histone H1 fusion protein, and even worse with histone H3 and H4 fusions (data not shown). Thus, we settled on H2B-mCherry as a nuclear marker for co-expression with NLS-GFP reporter genes.

### Monopartite cNLSs show weak nuclear targeting activity in *P. sojae*

To examine cNLS activity in *P. sojae*, three well-characterized monopartite cNLS sequences were individually fused to the N-terminus of 2XGFP and expressed in *P. sojae* transformants. To quantify the activities of different NLSs, the ratio of fluorescence intensity in nuclei compared to the cytoplasm (Nuc:Cyt) was measured within ~ 30 hyphae from at least three individual *P. sojae* transformants for each fusion. Due to the wide range of nuclear-cytoplasmic ratios observed, we found it convenient to express these ratios as log_2_(Nuc:Cyt), or LNC. As expected, 2XGFP alone was extensively localized to the cytoplasm (LNC = 0.52; Figures 1A,D). The well-studied cNLS derived from SV40 large T antigen (SV40 NLS) produced incomplete albeit statistically significant (FDR *p*′ < 0.001) nuclear localization in any fusion configuration (fusion at N- or C-terminus of 2XGFP) or copy number (LNC = 1.43 − 2.35; Figures 1A,B,D). In comparison, H2B-GFP exhibited an LNC of 7.94. Another monopartite cNLS prototype, the *c-Myc* NLS, produced similar results (LNC = 1.55; Figures 1A,D). To characterize cNLSs in native *P. sojae* nuclear proteins, we also analyzed eight protein fragments that contained monopartite cNLS motifs predicted by *PSORT II* (PSORT, 1997). As summarized in Table S3, we observed that none of the predicted monopartite cNLSs were sufficient for nuclear localization of GFP reporters.

### Functional bipartite cNLSs require additional basic amino acids compared to the conventional bipartite consensus

To examine the nuclear localization activities of bipartite cNLSs, the classic bipartite cNLS of nucleoplasmin (NPL) was assayed in *P. sojae*. This sequence produced strong nuclear localization (LNC = 5.33; Figures 1A,D). Small unstained regions in the centers of nuclei were validated as nucleoli by co-expression of the nucleolar marker, fibrillarin (from *Phytophthora capsici*; Genbank accession No. KY452016) fused to mCherry (Figure 1C). To explore the activity of bipartite cNLSs further, four protein segments that were predicted to contain bipartite cNLS sequences (by *PSORT II*) were identified from nuclear-localized *P. sojae* proteins, and assayed in *P. sojae* transformants. Of these four, two (PsH3_116−136_ and PHYSO_561151_505−521_) were incapable of producing strong localization of GFP reporters into *P. sojae* nuclei (Table S3). On the other hand, two regions carrying predicted bipartite cNLSs showed strong activity, namely one at the C-terminus of PHYSO_561151 and one at the N-terminus of PsL28. At the C-terminus of PHYSO_561151, the functional residues proved to be residues 504–520, displaced one position from the inactive predicted sequence at 505–521 (LNC = 4.68). At the N-terminus of PsL28, the functional residues appeared to be residues 7–27 (LNC = 3.59) (Figures 1E,F). Sequence comparisons of the functional bipartite cNLS sequences in PsL28 and PHYSO_561151 revealed the presence of additional arginine or lysine residues in the first of the two basic amino acid clusters that comprise the bipartite motif, compared to the canonical consensus developed from mammalian and yeast proteins (highlighted in red in Figure 1E). Mutation of these additional basic amino acids from the PsL28 or PHYSO_561151 bipartite NLSs showed that these residues were essential for the activity of these NLSs (Figures 1F,G). The human NPL bipartite cNLS however lacked any additional positive residues in the first basic cluster, but was functional. This cNLS has four consecutive lysines in the second cluster and two additional lysines between the two clusters; one or both of these features may make this cNLS functional in *P. sojae*. Although *PSORT II* did not accurately predict the functional bipartite NLSs in PsH3 and PsL28, both *NLStradamus* and *cNLS Mapper* identified broader regions that encompassed those extended NLSs (Tables S3, S4). Both programs also correctly predicted that PsH3_116−136_ would not be functional.

### Canonical PY-NLS motifs produce weak nuclear localization activity in *P. sojae*

**Figure 2:**
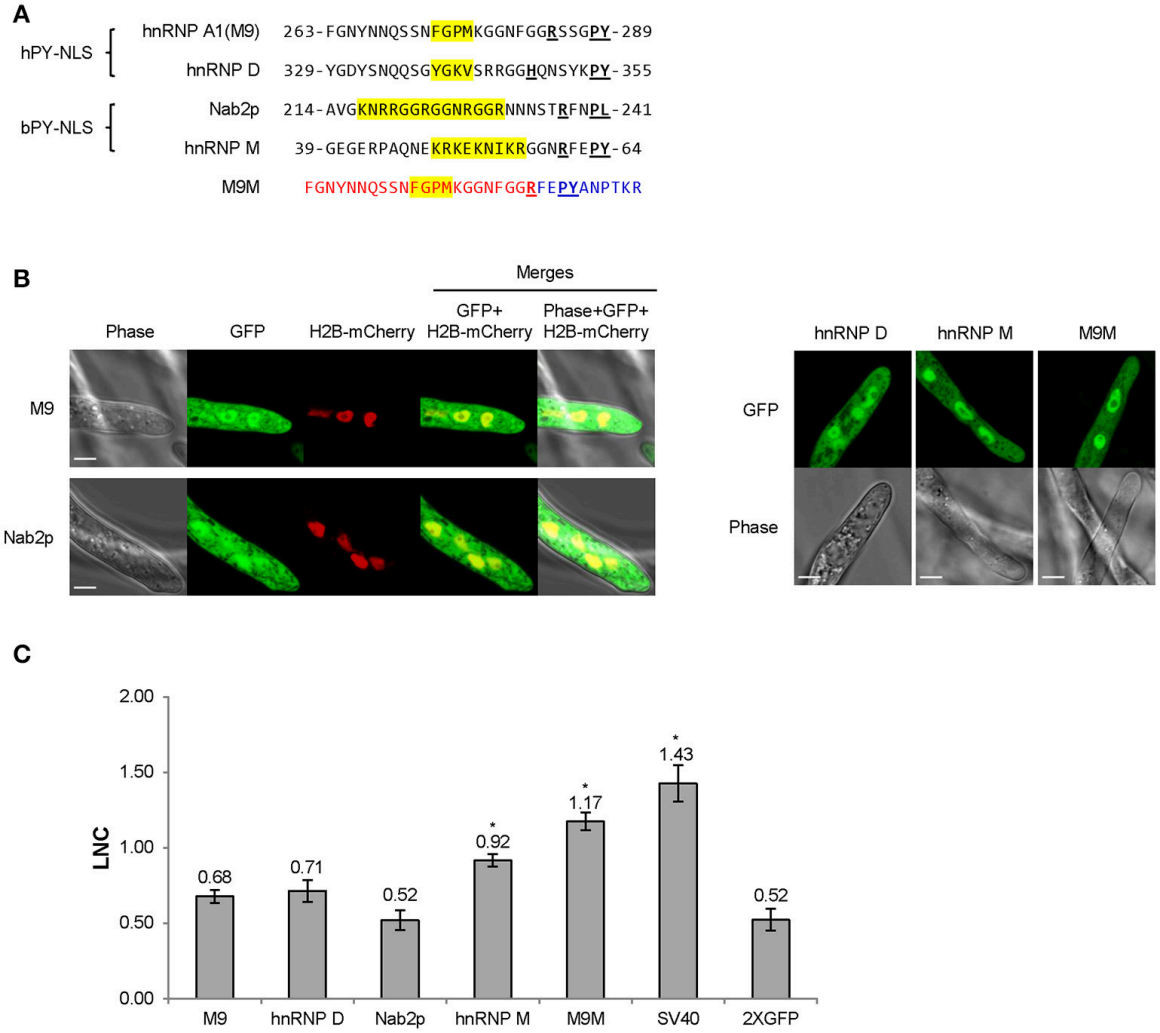
**PY-NLS prototypes exhibit weak nuclear targeting activities in *P. sojae* transformants. (A)** Sequences of four well-characterized PY-NLSs derived from human or yeast proteins and the Kapβ2-specific nuclear import inhibitor, M9M peptide. Core residues that determine hydrophobic or basic PY-NLS type are shaded in yellow. The R/K/H-X_2−5_-PY/L consensus residues are bold and underlined. Residues marked in red and blue in M9M are sequences originating from M9 and hnRNP M respectively. **(B)** Subcellular localization of the five PY-NLS described in **(A)**. Representative images are shown. **(C)** Quantification of localization of fusions observed in **(B)**. For ease of comparison, the LNC-values of SV40 NLS-2XGFP and 2XGFP alone (from Figure 1) are included in the bar chart here and in subsequent figures; i.e., the same values were used in every figure. Asterisks, LNC-values of NLS-2XGFP fusions that are significantly greater (FDR *p*′ < 0.001) than the LNC of 2XGFP.

Although a number of PY-NLS sequences have been characterized in human and yeast proteins, few of them have been reported in other organisms including oomycetes. To examine the activity of PY-NLS sequences in oomycetes, four well-characterized human and yeast PY-NLSs (two basic, bPY-NLS; two hydrophobic, hPY-NLS) were fused to the N-terminus of 2XGFP and their localization was examined in *P. sojae* transformants (Figure 2A). None of these PY-NLSs produced strong nuclear localization of the 2XGFP reporter (Figures 2B,C). Only the bPY-NLS in hnRNP M produced significantly more nuclear localization than 2XGFP alone (FDR *p*′ < 0.001) (Figures 2B,C). We also tested a synthetic peptide, M9M, that is a chimera of the hnRNP A1 (M9) hPY-NLS and the hnRNP M bPY-NLS (Figure 2A). This peptide has high affinity to the Kapβ2 PY-NLS binding site in human and is usually used as a Kapβ2-specific inhibitor (Cansizoglu et al., 2007). In *P. sojae* this peptide produced significantly more nuclear localization than the four PY-NLS prototypes (FDR *p*′ < 0.001), although the localization was still weaker than that produced by the SV40 NLS (Figures 2B,C).

**Figure 3:**
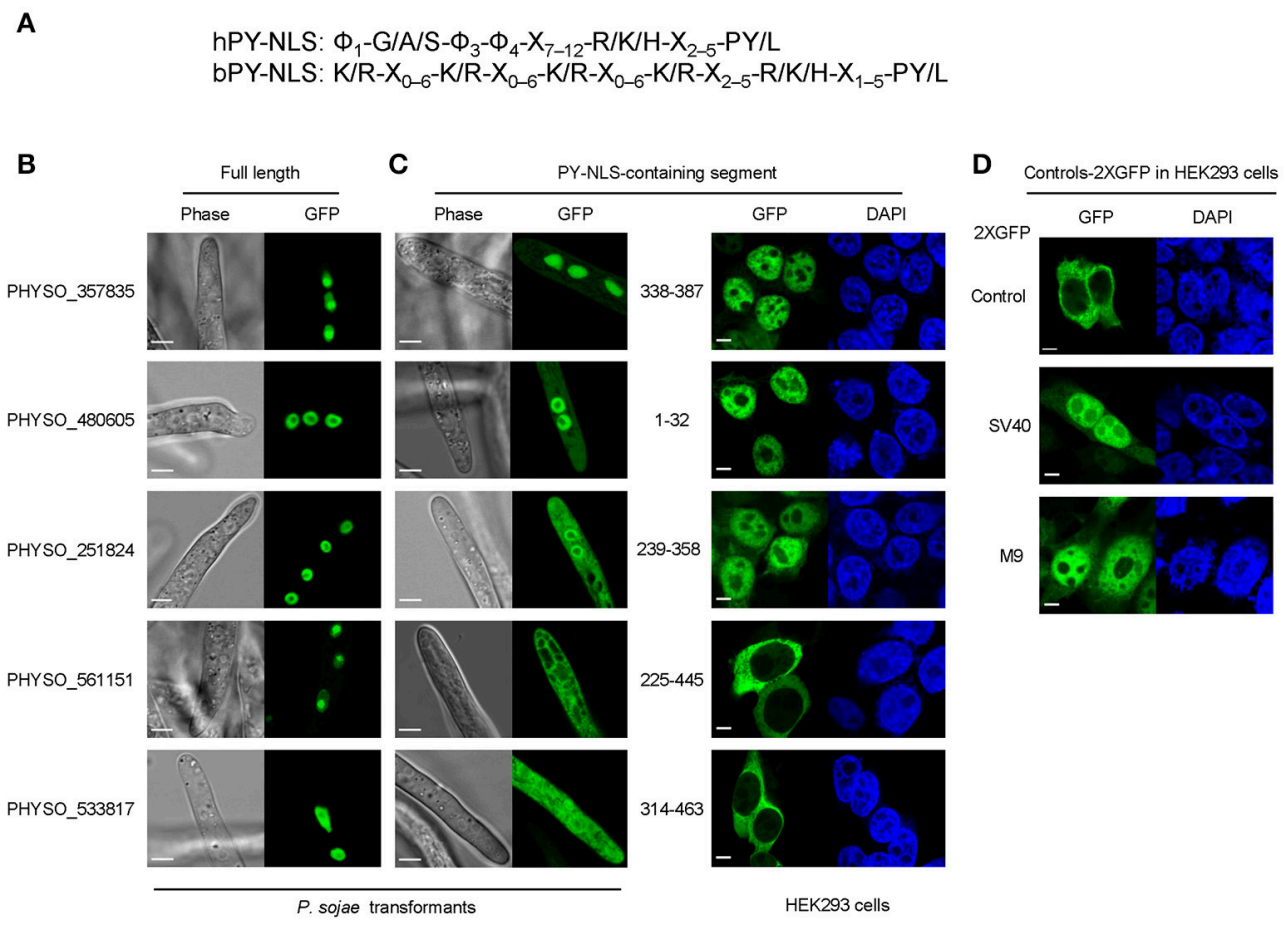
**Subcellular localization produced by five protein segments containing candidate PY-NLS motifs. (A)** The modified consensus sequence (Süel et al., 2008) used for searching for candidate PY-NLSs in *P. sojae*. Φ1 represents a hydrophobic residue (defined here as L, M, I, V, W, Y, H, A, P, or F) while Φ3 and Φ4 represent either hydrophobic residues or R or K./indicates alternative residues. X_m−n_ indicates any number of unspecified residues from m to n in number. **(B)** Nuclear localization of the full-length proteins PHYSO_357835, PHYSO_480605, PHYSO_251824, PHYSO_561151, and PHYSO_533817 in *P. sojae* transformants. Representative images. **(C)** Subcellular localization of PY-NLS-containing segments in *P. sojae* (left) and in HEK 293 cells (right). **(D)** HEK 293 cells expressing 2XGFP alone, SV40 NLS-2XGFP, and M9NLS-2XGFP, produced in the same experiment as controls. Representative images are shown in **(B–D)**.

Given that some prototypical PY-NLSs produced weak nuclear localization in *P. sojae*, we considered the possibility that a fully functional PY-NLS in *P. sojae* may require additional elements, as was the case for cNLSs. To search for functional PY-NLS sequences in *P. sojae* nuclear-localized proteins, we began by scanning the *P. sojae* proteome using the human/yeast PY-NLS consensus sequences (the “PL” rule was allowed for bPY-NLS, Figure 3A). Then we filtered for structural disorder and overall positive charge in the NLS. This two step scan revealed 25 proteins containing candidate hPY-NLSs and 200 containing candidate bPY-NLSs. Twelve candidates annotated as RNA-processing proteins or transcription factors were selected for further analysis.

We also extended the consensus-sequence-based search by including the “PL” option for hPY-NLSs. The rationale for this was that it was reported that the tyrosine in the C-terminal PY motif shows degeneracy in yeast (Süel et al., 2008). However, since yeast Kap104 only recognizes bPY-NLS motifs, the degeneracy of tyrosine in hPY-NLS was not clear. Thus, we reasoned that the “PL” rule for the bPY-NLSs may also apply to hPY-NLSs. By adding the “PL-rule” in the hPY-NLS search (Figure 3A), we obtained another 82 hPY-NLS candidates having C-terminal “PL” motifs. One candidate (PHYSO_480605, annotated as an mRNA maturation protein) was selected for further analysis because the putative PY-NLS was the only predicted NLS in the protein (Figure 3B). Another candidate, PHYSO_251824, had already been selected for testing because it also contained a separate conventional PY-NLS motif.

To validate the subcellular localization of the proteins, the 13 full-length proteins were tagged with GFP and expressed in *P. sojae* transformants. Five candidates showed strong nuclear localization in *P. sojae* hyphae at steady state, including PHYSO_480605 (Figure 3B and Table 1). PHYSO_561151 and PHYSO_357835 also showed nucleolar localization (Figure 3B). (Although PHYSO_561151 was initially selected as a PY-NLS candidate, its predicted PY-NLS proved to be inactive, as described below, and we subsequently determined that it contained a functional bipartite cNLS, as described above). Other candidates either could not be PCR amplified, contained incorrect intron annotations causing frame shifts, or appeared toxic when overexpressed in *P. sojae* transformants (data not shown).

**Table 1:** **Function of PY-NLSs predicted in *P. sojae* nuclear localized proteins**.

**FungiDB ID**	**Annotation**	**Core PY- NLS-like sequence^a^**	**Type^b^**	**Nuc^c^**
PHYSO_357835	U3 small nucleolar RNA—associated protein	359 *PAPA*DYTVATTRHK**R**IQ**PY** 377	h	√
PHYSO_480605	Cleavage and polyadenylation specificity factor subunit 3	1 *MSKR*RLAEEAADER**H**IMRIM**PL** 22	h, v	√
PHYSO_251824	mRNA cleavage and polyadenylation factor I complex, subunit RNA15	259 *PAPA*PAKSGGTRWSA**R**PG**PL** 278 332 *RDPRRAGRDPR*LA**K**R**PY** 348	h, v b	√ √
PHYSO_561151	Homeodomain-like transcription factor	329 *RGVEQQLKKVAVRADPK*RK**K**ELADV**PY** 355	b	×
PHYSO_533817	C2H2 zinc finger protein	333 *RTFKKEDARRQHQLAK*HG**K**D**PL** 354	b, v	×

a *Epitope 1 that determines the hydrophobic or basic sub-classes of PY-NLSs is highlighted in italics. The R/K/H-X_2−5_-PY/L motif is in bold and underlined*.

b *PY-NLS types: b, basic; h, hydrophobic; v, variant PL epitope*.

c *√ contributes to nuclear localization; ×, does not contribute*.

To determine whether the predicted PY-NLSs in the nuclear-localized proteins were sufficient to mediate nuclear localization of reporter proteins, protein segments containing the motifs were fused to 2XGFP at either the N- or C-terminus, based on their positions in the native proteins. One PY-NLS-containing segment, PHYSO_357835_338−387_, mediated very efficient nuclear localization (Figure 3C). Other candidates, namely PHYSO_480605_1−32_ and PHYSO_251824_239−358_, produced incomplete nuclear localization with some remaining cytoplasmic signals (Figure 3C). In contrast, the PY-NLS-containing segments PHYSO_561151_225−445_ and PHYSO_533817_314−463_ did not produce any nuclear localization (Figure 3C, Figures S2, S3). As noted above, nuclear localization of PHYSO_561151 subsequently turned out to be mediated by a bipartite cNLS at its C-terminus, while the nuclear localization of PHYSO_533817 was determined by an unidentified sequence between residues 172–314 (Figure S3).

Because four of the protein segments carrying candidate PY-NLSs showed weak or non-existent nuclear localization activity in *P. sojae*, it was unclear if the problem was the general reliability of the three PY-NLS prediction rules or whether the *P. sojae* import machinery did not efficiently utilize the predicted PY-NLS motifs. To address this question, parallel experiments were carried out in human embryonic kidney 293 cells (HEK 293) to determine the activity of the putative PY-NLSs in human cells. As shown in Figures 3C,D, the subcellular localization of the PY-NLS-containing protein segments in HEK 293 cells were well correlated with their localizations in *P. sojae*, except for segment PHYSO_480605_1−32_ that showed much stronger nuclear localization in the human cells.

To more precisely define the roles of the predicted PY-NLS motifs in the nuclear localization of each of PHYSO_357835, PHYSO_480605, and PHYSO_251824, a series of truncations and mutations were made. These analyses are detailed in the next three sections.

### An augmented PY-NLS sequence in PHYSO_357835 is necessary and sufficient for nuclear import

**Figure 4:**
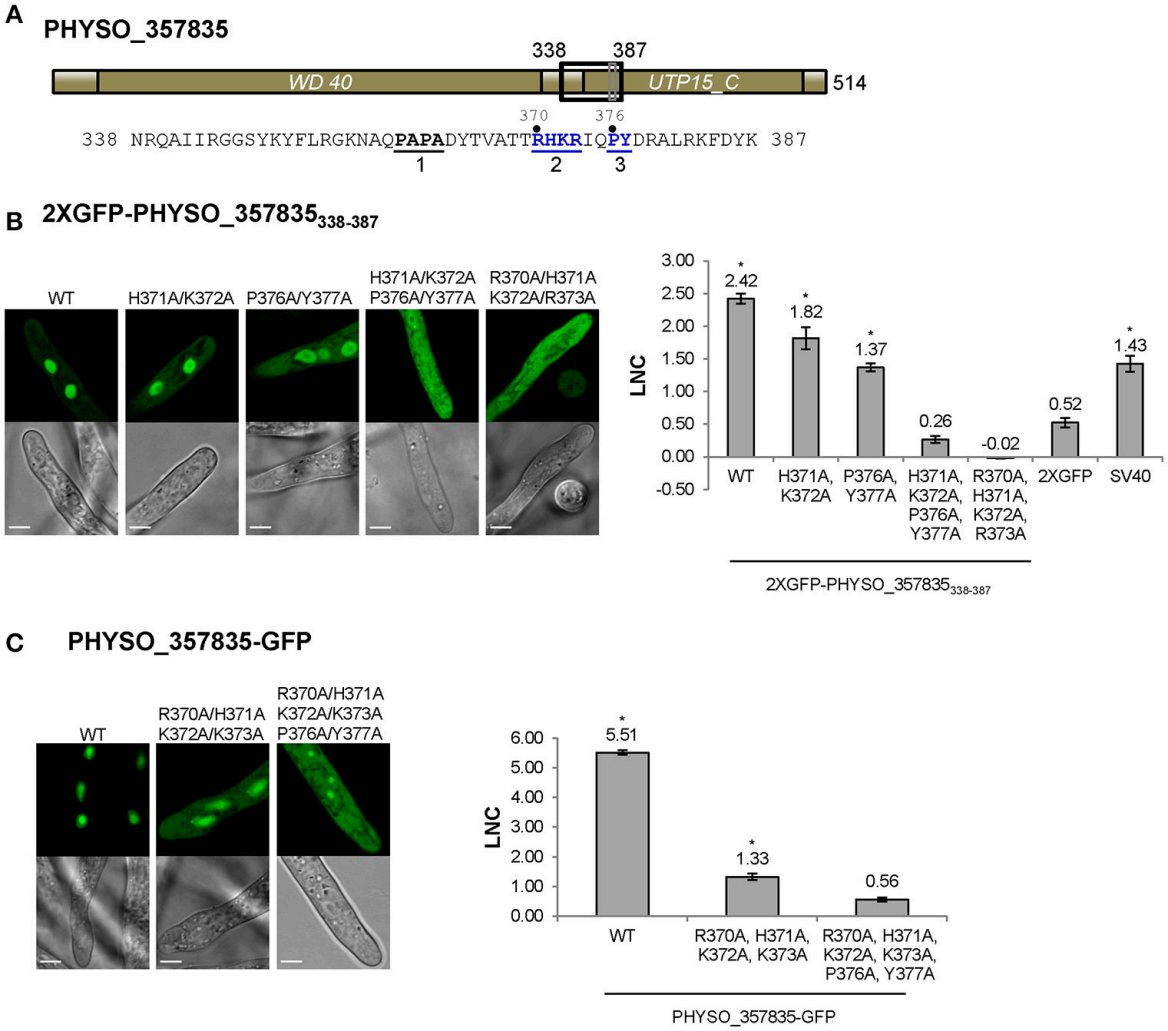
**Nuclear localization of PHYSO_357835 is mediated by a PY-NLS that incorporates a cNLS-like motif. (A)** Domain structure of PHYSO_357835. The position of the PY-NLS and the predicted cNLS within PHYSO_357835 are indicated by a black and a gray rectangle, respectively, and the corresponding amino acid sequence is listed below. The three PY-NLS epitopes are in bold, underlined, and numbered. Blue residues indicate the PY motif and basic region corresponding to the predicted cNLS, which were subjected to mutational analysis. No NLS sequences were predicted by *NLStradamus* or *cNLS Mapper*. **(B)** Subcellular localization of PHYSO_357835 mutants in the context of the C-terminal domain, 338–387. Left, representative images from *P. sojae* transformants expressing various mutations of 2XGFP-PHYSO_357835_338−387_. Right, quantification of the localization of 2XGFP-PHYSO_357835_338−387_ fusion proteins. **(C)** Subcellular localization of PHYSO_357835 mutants in the context of full length PHYSO_357835-GFP. Left, representative images; right, quantitation. The dots observed in *P. sojae* hyphae expressing PHYSO_357835-GFP-(R370A/H371A/K372A/R373A/P376A/Y377A) may be nucleoli as the WT shows substantial nucleolar localization, but this was not verified. The LNC for this mutant was calculated assuming the dots were nucleoli. Asterisks, LNC-values of NLS-2XGFP fusions that are significantly greater (FDR *p*′ < 0.001) than the LNC of 2XGFP.

The aforementioned experiments indicated that residues 338–387 of PHYSO_357835, containing a predicted PY-NLS sequence, were sufficient to mediate localization of the 2XGFP reporter into the *P. sojae* nucleus. However, examination of the PHYSO_357835 protein sequence using *PSORT II* revealed the presence of another candidate NLS within this segment, namely a monopartite cNLS-like sequence (370-RHKR-373) overlapping with the epitope 2 of the PY-NLS motif (Figure 4A). To test whether nuclear localization by PHYSO_357835_338−387_ required the putative cNLS separately from the epitope 2 of the PY-NLS-like sequence, histidine at 371 and lysine at 372 were mutated to alanines, resulting in 370-RAAR-373 (the canonical PY-NLS consensus requires only a single basic residue at this position). Although 2XGFP-PHYSO_357835_338−387_ (H371A/K372A) appeared slightly more cytoplasmic than wild type (LNC = 1.82 compared to 2.42), the reporter remained primarily nuclear, suggesting that the nuclear localization of PHYSO_357835_338−387_ was not primarily dependent on this putative cNLS.

To confirm whether the predicted PY-NLS sequence within PHYSO_357835_338−387_ was the only NLS that determined the nuclear localization of PHYSO_357835_338−387_, amino acid substitutions were introduced at the key residues in the predicted PY-NLS sequence. As shown in Figure 4B, mutation of the PY residues (P376A/Y377A) reduced but did not eliminate nuclear localization (LNC = 1.37). However, when the cNLS mutations H371A/K372A were combined with the PY mutations, nuclear localization produced by PHYSO_357835_338−387_ was abolished (LNC = 0.26; Figure 4B), suggesting that the PY motif, in combination with the cNLS, is required for the nuclear localization of PHYSO_357835_338−387_. To further explore the role of the basic region (370-RHKR-373), all the four basic residues were converted to alanines. This mutation also abolished nuclear localization (LNC = −0.02), indicating that the full set of four residues of this basic region is essential for nuclear import. Together these results suggest that both the predicted PY-NLS (defined by the residues PY in combination with at least 1 of RHKR) and the predicted cNLS (defined by all four of RHKR) are required for efficient nuclear localization of this *P. sojae* protein.

To test the role of this cNLS-augmented PY-NLS sequence in import of the full length PHYSO_357835 protein, mutations in the basic region (R370A/H371A/K372A/R373A) alone and combination with the PY dipeptide (R370A/H371A/K372A/R373A/P376A/Y377A) were introduced into full-length GFP-tagged protein. Mutation of the basic region resulted in substantial mislocalization of the full length protein into the cytoplasm (from LNC of 5.51 to 1.33), and additional mutation of the PY motif further decreased the LNC-value (to 0.56, not significantly different than 2XGFP alone; FDR *p*′ = 0.7; Figure 4C). Together, these results indicate that the augmented PY-NLS sequence of PHYSO_357835 is necessary as well as sufficient for the nuclear localization of this protein. Other than *PSORT II*, which predicted the RHKR element of this NLS, no current software programs, including *NLStradamus* or *cNLS Mapper*, predicted the functional NLS sequences of PHYSO_357835 (Tables S3, S4).

### Nuclear import of PHYSO_480605 is mediated by a variant PY-NLS

**Figure 5:**
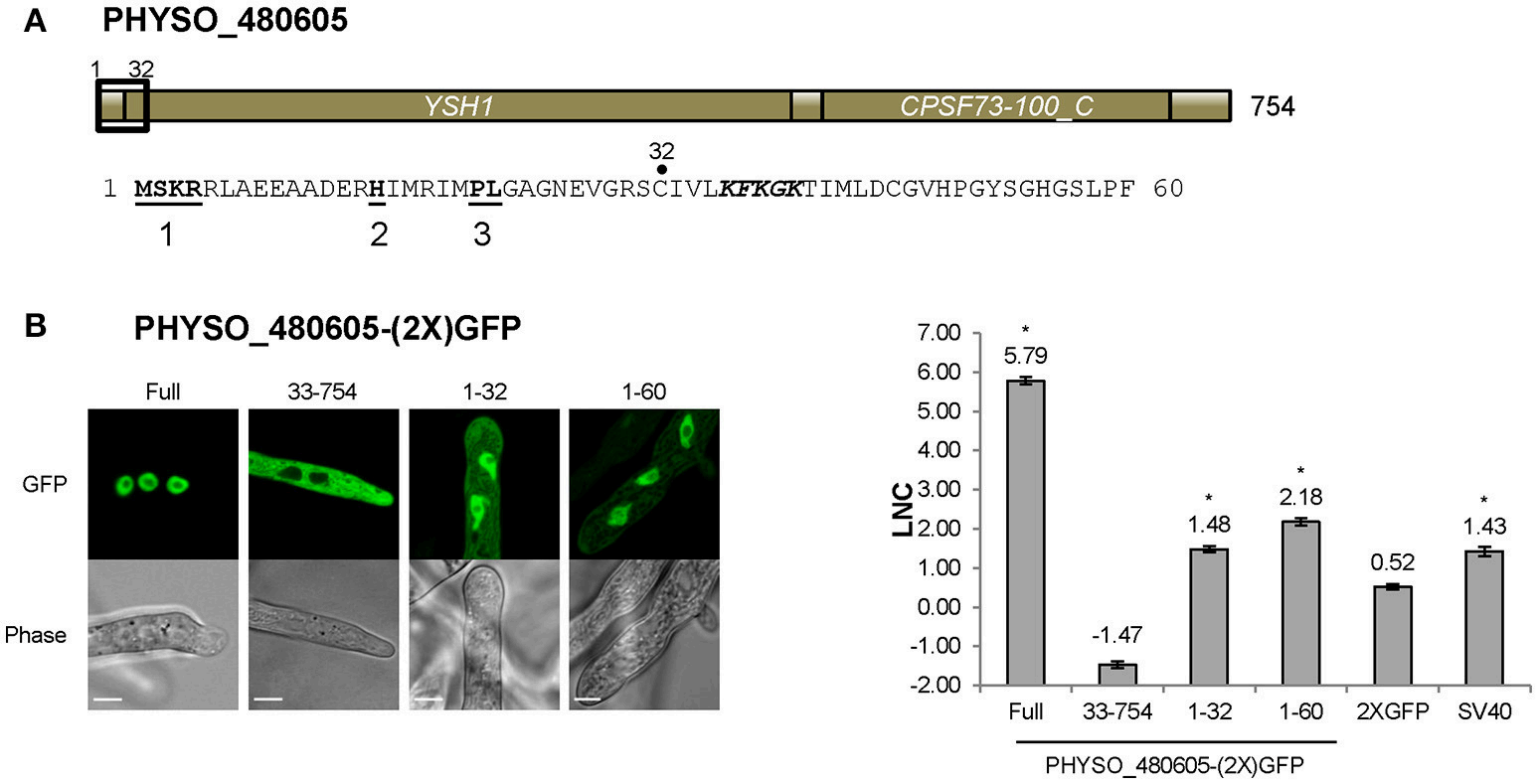
**Nuclear localization of PHYSO_480605 requires a region containing a PY-NLS with a variant PY motif. (A)** Domain structure of PHYSO_480605. Position of the predicted PY-NLS is indicated by a black rectangle and the corresponding sequence is listed below. The three PY-NLS epitopes are in bold and underlined. The basic patch corresponding to a predicted cNLS is in bold and italics. No NLS sequences were predicted by *PSORT II, NLStradamus* or *cNLS Mapper*. **(B)** Subcellular localization of 2XGFP with full length PHYSO_480605 or fragments of it in *P. sojae* transformants. Left, representative images; right, quantification. Asterisks, LNC-values of NLS-(2X)GFP fusions that are significantly greater (FDR *p*′ < 0.001) than the LNC 2XGFP.

As noted above, the predicted PY-NLS located at 1–32 within PHYSO_480605 contains terminal PL residues rather than PY residues. To test if this variant motif is required for PHYSO_480605 nuclear localization, the protein was split at position 32 and each fragment was fused to 2XGFP and expressed in *P. sojae*. The N-terminal 32 residues fused to 2XGFP exhibited significant nuclear localization with some visible cytoplasmic signal (LNC = 1.48, FDR *p*′ < 0.001; Figures 2B, 5B), while the C-terminal fragment (residues 33–754) was exclusively distributed in the cytoplasm (Figure 5B). These results indicated that the fragment containing the variant PY-NLS was necessary for the nuclear import of PHYSO_480605, but suggested that additional amino acids downstream of the motif may contribute to the strength of the nuclear targeting (LNC of the full length protein was 5.79). In fact, an expanded fragment (1–60) that includes a stretch of positively charged amino acids (36-KFKGK-40) showed significantly increased nuclear localization (from LNC = 1.48 to 2.18, FDR *p*′ < 0.001; Figure 5B). However, localization was still much less than the full length protein (LNC = 5.79), suggesting that additional downstream sequences might augment nuclear localization, despite being insufficient to independently direct localization. No current software programs, including *PSORT II, NLStradamus* or *cNLS Mapper*, predicted any NLS sequences in PHYSO_357835 (Tables S3, S4).

### Nuclear localization of PHYSO_251824 requires collaboration of three distinct NLS-like sequences within the C-terminus

**Figure 6:**
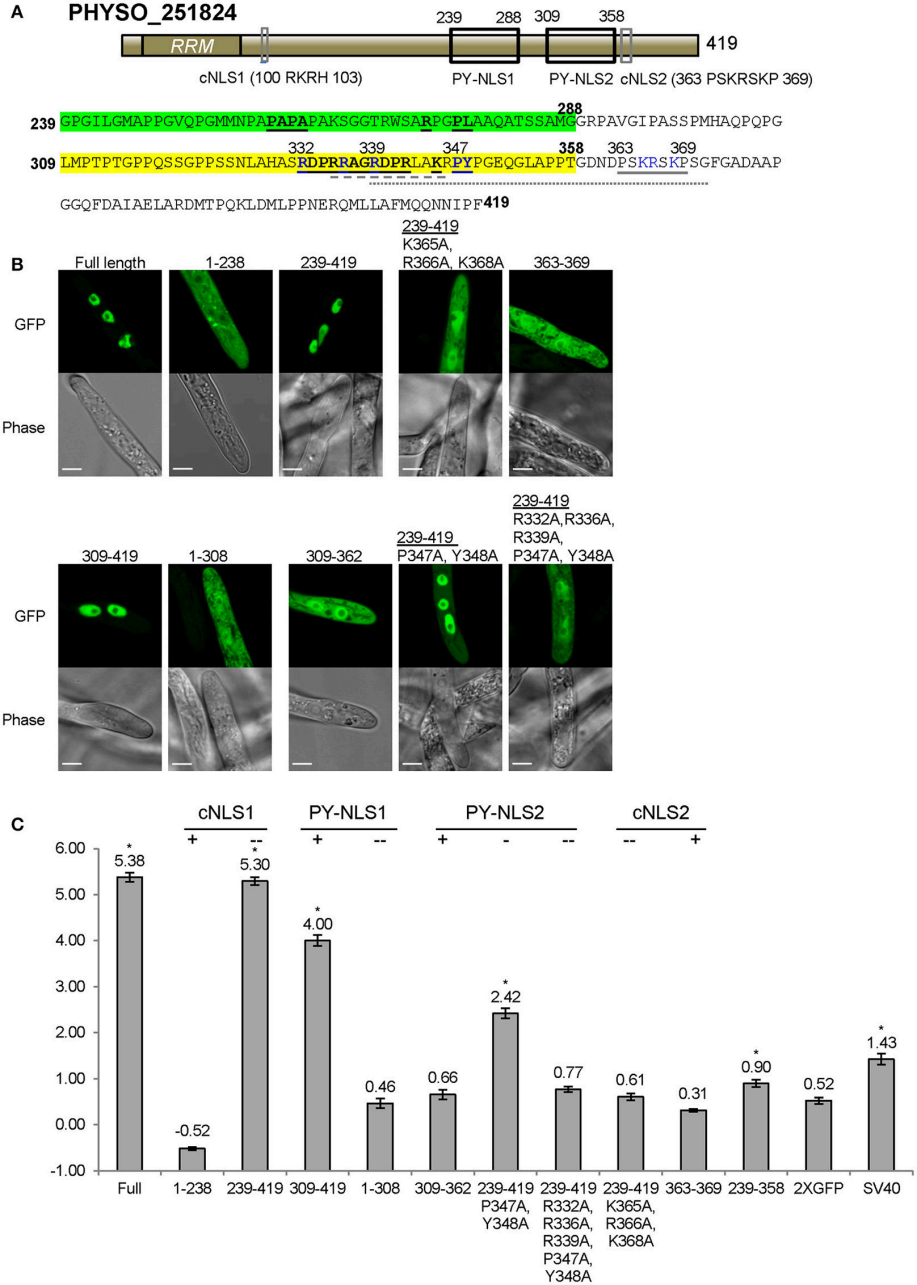
**Nuclear localization of PHYSO_251824 requires contributions from two PY-NLS and one cNLS clustered within the C-terminus. (A)** Domain structure of PHYSO_251824. The two candidate PY-NLSs and the two *PSORT II*-predicted cNLSs are indicated by black and gray rectangles respectively; their corresponding sequences are shown below. Predicted cNLS1 is inactive. The three epitopes of the two PY-NLS sequences are in bold and underlined. Amino acids subjected to mutational analysis are in blue. Amino acid sequences underlined by gray solid, dash, and dotted lines indicate the NLSs predicted by *PSORT II, NLStradamus*, and *cNLS Mapper* respectively. **(B)** Subcellular localization of PHYSO_251824-GFP and mutants fused to 2XGFP in *P. sojae* transformants. N-terminal truncations and cNLS2 were fused to 2XGFP at their C-termini, while various C-terminal PHYSO_251824 truncations were fused to 2XGFP at their N-termini. Representative images are shown. **(C)** Quantification of localization of PHYSO_251824 mutants. Image of PHYSO_251824_239−358_ is shown in Figure 3C. Mutational statuses of the various NLS candidates are labeled at the top. +, NLS candidate is the only one in the segment; -, NLS candidate is partially mutated; - -, NLS candidate is completely mutated. Asterisks, LNC-values of NLS-2XGFP fusions that are significantly greater (FDR *p*′ < 0.001) than the LNC of 2XGFP.

As noted above, residues 239–358 of PHYSO_251824 produced only weak nuclear localization (LNC = 0.90, FDR *p*′ < 0.002; Figures 3C, 6C). To better identify the sequences required for localization, we generated fusions containing larger protein fragments of PHYSO_251824. The entire N-terminal (1–238) and C-terminal (239–419) segments of PHYSO_251824 were expressed as fusions with 2XGFP in *P. sojae* transformants. PHYSO_251824_1−238_-2XGFP showed strictly cytoplasmic distribution (LNC = −0.52), despite possessing a putative monopartite cNLS (cNLS1, 100-RKRH-103) (Figures 6A,B). In contrast, 2XGFP-PHYSO_251824_239−419_ produced predominant nuclear localization (LNC = 5.30 compared to 5.38 for the full length protein). Comparison of the localization of 2XGFP-PHYSO_251824_239−419_ (LNC = 5.30) and 2XGFP-PHYSO_251824_239−358_ (LNC = 0.90) indicated that residues 359–419 must contribute to NLS function (Figure 6C). Examination of those residues using *PSORT II* revealed another predicted monopartite cNLS (363-PSKRSKP-369, cNLS2) (Figure 6A). To test whether cNLS2 was necessary for nuclear localization produced by PHYSO_251824_239−419_, the basic amino acids in cNLS2 were all substituted with alanines (K365A/R366A/K368A); this mutation dramatically reduced the nuclear localization of 2XGFP-PHYSO_251824_239−419_ (LNC = 0.61 vs. 5.30; Figures 6B,C). To test whether cNLS2 was sufficient for nuclear localization, residues 363–369 were fused to 2XGFP; however these residues alone were not sufficient to direct 2XGFP into the nucleus (LNC = 0.31; Figures 6B,C). Thus, cNLS2 was revealed to be a non-autonomous enhancer of nuclear localization by PHYSO_251824_239−419_.

In addition to cNLS2, residues 239–419 of PHYSO_251824 contain two predicted PY-NLS motifs. PY-NLS1 is located at positions 239–288 and has a variant PY motif (PL), while PY-NLS2 is a conventional PY-NLS located 309–358 (Figure 6A). To test the contribution of PY-NLS1, residues 239–308 were deleted. The resulting segment, 2XGFP-PHYSO_251824_309−419_ showed a significantly reduced localization compared to 2XGFP-PHYSO_251824_239−419_(LNC = 4.00 vs. 5.30, FDR *p*′ < 0.001; Figure 6C). This finding suggested that PY-NLS1 may have weak NLS activity. In support of this conclusion, extension of residues 239–308 onto PHYSO_251824_1−238_-2XGFP resulted in the reappearance of some nuclear localization (LNC = 0.46 vs. −0.36, FDR *p*′ < 0.001; Figures 6B,C). To address the contribution of PY-NLS2, specific amino acids were converted into alanines in the PY-NLS2. In the context of residues 239–419, substitution of the PY residues of PY-NLS2 to alanines (P347A/Y348A) partially reduced the nuclear localization (from LNC = 5.30 to 2.42, FDR *p*′ < 0.001), while additional substitutions in the basic epitope (R332A/R336A/R339A/P347A/Y348A) effectively eliminated the nuclear localization (LNC of 0.77, not significantly greater than 2XGFP alone; Figures 6B,C). On the other hand, the PY-NLS2 alone (309–362) was not sufficient to direct 2XGFP into the nucleus (LNC = 0.66; Figures 6B,C), suggesting that it is necessary but not sufficient for efficient nuclear localization.

Taken together, these findings indicate the nuclear localization of PHYSO_251824 is determined by the C-terminal region, in which two weak PY-NLSs and one cNLS-like enhancer sequence operate synergistically to this protein into the nucleus. No current software programs, including *PSORT II, NLStradamus* or *cNLS Mapper*, fully predicted the functional NLS sequences of PHYSO_251824 (Tables S3, S4). *PSORT II* predicted cNLS2, *NLStradamus* predicted a fragment of PY-NLS2, while *cNLS Mapper* predicted a fragment of PY-NLS2 in combination with cNLS2.

### Highly conserved nuclear-localized proteins show different sequence requirements for nuclear import in *P. sojae* than in human and yeast counterparts

To examine whether *P. sojae* utilizes the same nuclear import sequences for transport of conserved nuclear-localized proteins, we examined ribosomal proteins and core histones. Newly synthesized ribosomal proteins are transported into the nucleus in order to assemble with rRNAs in the nucleolus (Lafontaine and Tollervey, 2001; Gerhardy et al., 2014). Histones, including H2A, H2B, H3, H4, together with the linker histone H1, are essential components of chromatin (Baake et al., 2001).

**Figure 7:**
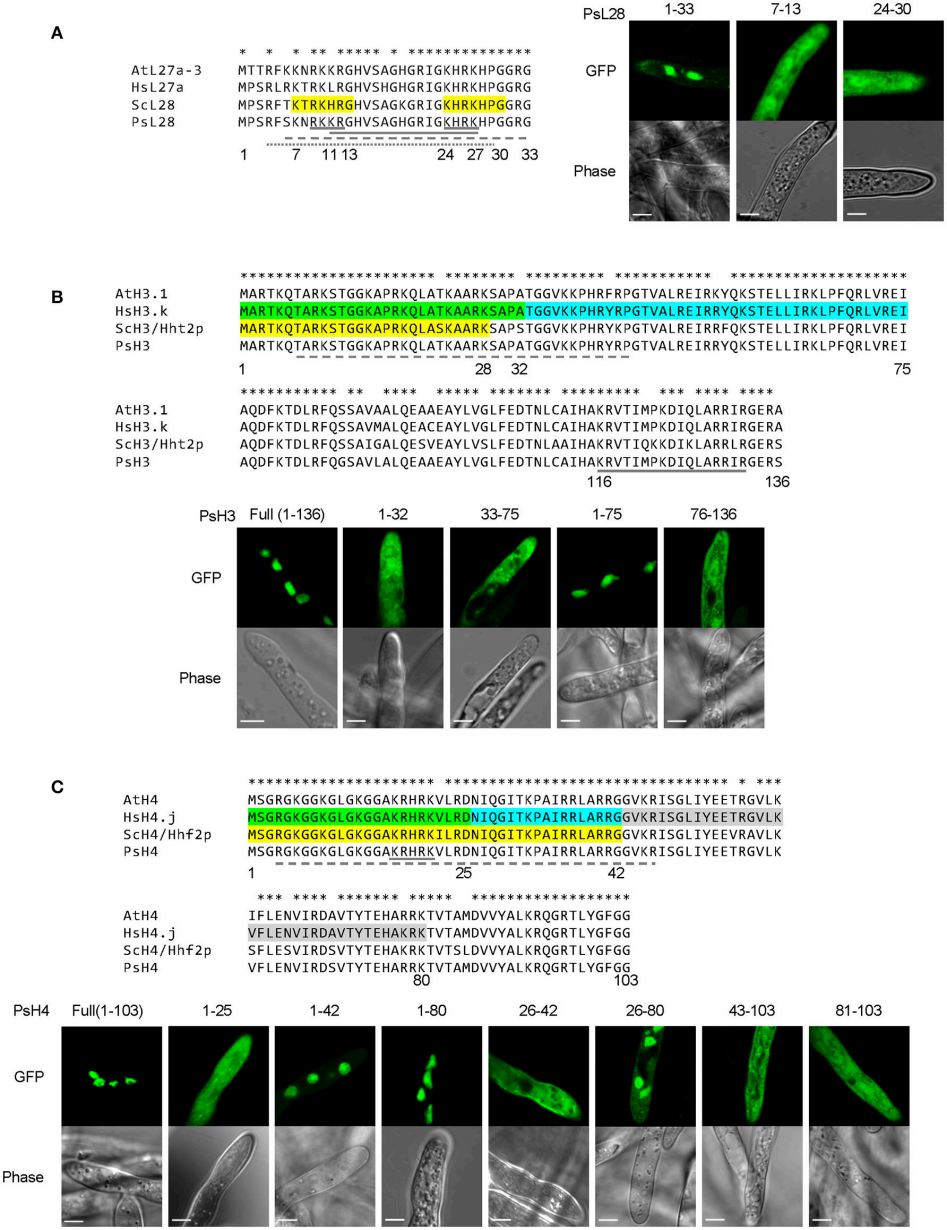
**Combinatorial usage of NLSs for nuclear transport of *P. sojae* ribosomal protein L28 and core histones H3 and H4**. **(A–C)** Upper panels, alignments of *P. sojae* ribosomal proteins L28 (PsL28, PHYSO_355737) and core histones H3 (PsH3, PHYSO_286415) and H4 (PsH4, PHYSO_285922) with their *Arabidopsi*s *thaliana* (At), human (Hs), and *Saccharomyces cerevisiae* (Sc) orthologs. Asterisks on the top of each alignment indicate conserved residues among L28, H3, or H4 orthologs. Regions individually conferring nuclear targeting in human core histones are highlighted in green, cyan, or gray while those required in yeast are highlighted in yellow. NLSs predicted in the *P. sojae* core histone and ribosomal protein by *PSORT II, NLStradamus*, and *NLS Mapper* are underlined by gray solid, dash, and dotted lines respectively. Lower panels, subcellular localization of various PsL28, PsH3, or PsH4 truncations. Representative images are shown.

We first examined the *P. sojae* ribosomal protein PsL28 which is the ortholog of the yeast ribosomal protein L28 (ScL28, former name L29, Underwood and Fried, 1990; the naming system for *P. sojae* ribosomal proteins follows the nomenclature of *S. cerevisiae*; Mager et al., 1997). Protein sequence alignment revealed that the N-termini of the L28 orthologs are highly conserved among different organisms (Figure 7A). Based on functional assays, ScL28 was reported to contain two NLSs: ScL28-NLS1, located at amino acid residues 7–13, and ScL28-NLS2 at 24–30 (Underwood and Fried, 1990). The sequence corresponding to NLS1 in PsL28 showed two amino acid differences from ScL28-NLS1, while the NLS2 sequence was exactly the same in all L28 orthologs (Figure 7A). The minimal conserved region of PsL28_1−33_ that contains both NLSs was expressed as a fusion with 2XGFP and showed strong nuclear localization (Figure 7A). In contrast, when each putative NLS was tested separately by fusion with 2XGFP, neither one produced nuclear localization in *P. sojae* transformants (Figure 7A). However, as indicated above (Figures 1E,F), the two sequences in PsL28 corresponding to NLS1 and NLS2 together constitute an extended bipartite NLS that includes a core bipartite NLS motif (residues 11–27) together with additional flanking positive residues at the N-terminus; this extended bipartite NLS is functional even though the core bipartite NLS [PsL28_1−33_(K7A/R9A/K10A)] is not (Figures 1E,F). Both *NLStradamus* and *cNLS mapper* correctly predicted this extended bipartite cNLS (Tables S3, S4).

We also tested two other ribosomal proteins, S22a and L3. Segments 21–29 of *P. sojae* S22a (PsS22a) and 1–22 of *P. sojae* L3 (PsL3), contain residues that are nearly identical to sequences that produced nuclear localization in yeast (Moreland et al., 1985; Timmers et al., 1999). However, in *P. sojae* transformants these segments produced only cytoplasmic accumulation (Figure S4); addition of flanking sequences (PsS22a_1−34_ and PsL3_1−36_) had no effect on nuclear localization either. Thus, other or additional sequences appear to be required for nuclear localization of these proteins in *P. sojae*. *PSORT II* and *NLStradamus* incorrectly predicted PsL3_1−36_ should be nuclear localized, but *cNLS Mapper* did not. All three predicted that PsS22a_1−34_ would not be nuclear localized and *PSORT II* predicted the presence of a cNLS motif elsewhere in the protein (117-RRKH-120) (Table S4).

A similar strategy was carried out to determine the activities of putative NLSs in core histones H3 and H4, which are nearly identical in amino acid sequences in all eukaryotes (Figures 7B,C). The NLSs in H3 and H4 have been experimentally characterized in human and yeast (Figures 7B,C). There is only one NLS reported in each of yeast H3 (ScH3) and H4 (ScH4) (Mosammaparast et al., 2002), while more than one NLS has been found in their human counterparts (Baake et al., 2001). The NLSs in ScH3 and ScH4 somewhat overlapped with their human orthologs, suggesting a common origin.

A series of truncations were made of *P. sojae* H3 (PsH3) and H4 (PsH4) according to the NLS locations in the yeast and human orthologs. The resulting fragments were tested for nuclear localization as 2XGFP fusions. As expected, the full-length PsH3 and PsH4 reporter constructs produced strong nuclear localization (Figures 7B,C). However, none of the H3 fragments responsible for nuclear localization in yeast or human (residues 1–32 or residues 33–75) individually exhibited the same NLS activities in *P. sojae* transformants. Region 1–75 of PsH3, which encompasses both of the two NLS regions of human H3, did produce strong nuclear localization in *P. sojae* (Figure 7B). Expression of a *P. sojae* H3 segment that lacked the N-terminal domain, H3_76−136_, did not produce nuclear localization (Figure 7B), indicating that no other sequences contribute to PsH3 nuclear localization. *NLStradamus* predicted part of this region (10–36) but not all of it. *PSORT II* and *cNLS mapper* did not predict this region (Tables S3, S4).

In the case of PsH4, residues 1–25, which produced partial NLS activity in yeast (Mosammaparast et al., 2002) and full activity in human cells (Baake et al., 2001), produced no observable nuclear localization in *P. sojae* (Figure 7C). A larger N-terminal GFP fusion PsH4_1−42_-2XGFP produced clear nuclear localization in *P. sojae* with little detectable fluorescence in the cytoplasm, while residues 1–80 of PsH4 produced complete nuclear localization (Figure 7C). This result suggests that segment 26–42 of PsH4, which was identified as an NLS in human cells, contains determinants that may contribute to NLS function in *P. sojae*. However, PsH4_26−42_ alone was insufficient to mediate nuclear localization of the GFP reporter, indicating that these residues could not act as an independent NLS (Figure 7C). On the other hand, PsH4_26−80_, which spans two regions (26–42 and 43–80) with NLS activity in human, did produced some nuclear localization in *P. sojae* but less than 1–80 (Figure 7C), whereas 43–103 showed no activity (Figure 7C). Together, these results suggest that all three regions corresponding to NLSs in human H4, namely 1–25, 26–42, and 43–80, must work together in *P. sojae* to produce efficient nuclear localization. *P. sojae* H4 reporters that lacked the N-terminal domains (H4_81−103_) produced no nuclear accumulation (Figure 7C). *NLStradamus* predicted the first two of these regions, but not the third. *PSORT II* and *cNLS Mapper* predicted none of them (Tables S3, S4).

Taken together, the results above suggest that compared to their yeast or human counterparts, *P. sojae* has a similar but stronger set of sequence requirements for translocation of conserved nuclear proteins into the nucleus.

## Discussion

The diverse sequences and structures of NLSs have limited the reliable prediction of nuclear-localized proteins based on amino acid sequence alone. Though consensus sequences have been proposed for some NLS types, such as cNLS and PY-NLS, their application remains constrained by many factors, such as protein context and flanking sequences, and by nuclear import regulation by mechanisms such as phosphorylation and protein interactions (Garcia-Bustos et al., 1991). Moreover, the karyopherins that bind NLSs show differences in specificity from species to species (Marfori et al., 2011), limiting the reliability of NLS consensus sequences when applied to widely divergent species. In this study, after eliminating artifacts caused by staining with DAPI, we identified key differences in several classes of NLSs between the oomycete *P. sojae* on the one hand, and human and yeast on the other hand.

### *P. sojae* classical NLSs exhibit distinct differences from human and yeast

The cNLSs are the best-characterized class of NLSs to date (Lange et al., 2007). The prototypical monopartite cNLS derived from the SV40 large T antigen (SV40 NLS) has been reported to direct efficient nuclear import in various organisms, including yeast, mammalian cells (Lange et al., 2007), and plants (Chang et al., 2012). However, we found that monopartite cNLSs, represented by the SV40 NLS and by the one from the *c-Myc* protein, produced relatively weak nuclear localization in *P. sojae*. It has been reported that protein context may influence the activity of NLSs, and multiple copies of a NLS can produce faster, more pronounced nuclear localization (Garcia-Bustos et al., 1991). We showed that SV40 NLS fusions at the C-terminus of GFP, and multiple copies of the NLS increased nuclear accumulation compared to a single copy at the N-terminus of GFP (Figure 1D), although some fluorescence was still visible in the cytoplasm (Figure 1B).

Based on assessment of several putative cNLSs predicted from the consensus sequences in *P. sojae* nuclear proteins, we found that cNLS-mediated nuclear import is conserved in *P. sojae* but that it exhibits some consistent differences: (1) most predicted monopartite cNLSs are not sufficient by themselves for efficient nuclear accumulation of GFP reporters. However, they may function to augment the activity of other NLSs or work collectively with other weak NLSs to accomplish efficient nuclear import of proteins. For example, the monopartite cNLS embedded inside the PY-NLS of PHYSO_357835 was essential for efficient nuclear targeting (Figure 4), while in PHYSO_251824 a C-terminal monopartite cNLS cooperated with two PY-NLSs to direct efficient nuclear localization (Figure 6). (2) With regard to bipartite cNLSs, sequence comparisons and mutational analyses of several functional bipartite cNLSs, including nucleoplasmin, PsL28, and PHYSO_561151, revealed that a functional bipartite cNLS in *P. sojae* typically requires additional basic amino acids in the first sub-motif, compared to the human/yeast consensus and possibly in the second also (Figures 1E,G). We did not test the contributions of non-positively charged residues in the neighborhood of the two positively charged clusters, but there is evidence that flanking residues also may be important for NLS activity (Kosugi et al., 2009; Lange et al., 2010).

### Efficient PY-NLS-mediated nuclear import requires additional clusters of basic amino acids

None of the human and yeast PY-NLSs we tested were capable of mediating efficient nuclear entry of GFP reporters in *P. sojae*, though weak NLS activity was observed with the hnRNP M PY-NLS and the chimeric M9M peptide. Given that the M9M peptide is a hPY-NLS (Figure 2A), our findings suggest that, in contrast to yeast, the *P. sojae* nuclear transport machinery may have evolved to transport both bPY-NLS and hPY-NLS cargos.

To characterize *P. sojae* PY-NLSs, we analyzed five native *P. sojae* nuclear-localized proteins which harbored predicted PY-NLS-like motifs. Using deletion and substitution mutations, we identified PY-NLS-containing fragments from three of the proteins that could mediate different degrees of GFP translocation into the nuclei of both *P. sojae* and mammalian cells. In each case however, additional basic amino acids were required, either in association with one of the three PY-NLS epitopes (as in PHYSO_357835), or C-terminal to the PY-NLS motif (PHYSO_480605 and PHYSO_251824).

In yeast, Süel et al. (2008) proposed the “degeneracy rule” for the bPY-NLS in which PL is exchangeable with PY. However, yeast Kapβ2 only recognizes the basic class of PY-NLSs, so PY-motif degeneracy remains unclear for the hydrophobic class of PY-NLSs. Only a few PY-NLSs are reported to have a variant PY motif, such as the RNA-binding protein HuR which contains a PG motif instead of PY. In addition, several newly identified Kapβ2/Kap104 cargos were reported to lack a typical PY motif or even a recognizable PY-NLS (reviewed in Soniat and Chook, 2015). In our study, we found that the variant “PL”-NLS in PHYSO_480605 produced substantial nuclear localization in both *P. sojae* and mammalian cells, suggesting the degeneracy rule may also exist for hPY-NLS.

The PY-NLS “consensus sequence” is defined by a collection of three modular epitopes: an N-terminal hydrophobic or basic residue-enriched motif, a second R/K/H motif and the third P[Y/L] motif (Lee et al., 2006; Süel et al., 2008). Biochemical and biophysical analyses have shown that in different NLSs each epitope may contribute differently to Kapβ2 (or Kap104p) binding (Lee et al., 2006; Cansizoglu et al., 2007; Süel et al., 2008). For instance, in hnRNP M and in the yeast mRNA processing protein Hrp1, epitope 3 contributes significantly more than the other two epitopes (Cansizoglu et al., 2007; Süel et al., 2008), In contrast, in the M9NLS and Nab2p PY-NLS, epitope 3 contributes weakly to Kapβ2 (or Kap104p) binding, while strong binding is conferred by epitope 1 or by multiple positions distributed across the three epitopes (Lee et al., 2006; Süel et al., 2008). In our study, we found that mutation of PY (epitope 3) in the PHYSO_357835 PY-NLS sequence reduced but did not abolish nuclear entry, whereas complete abrogation of nuclear entry was observed upon mutation of the basic cluster overlapping epitope 2. In the case of the PY-NLSs in PHYSO_480605 and PHYSO_251824, an extra basic cluster downstream of the PY-NLS consensus markedly enhanced the nuclear entry, effectively constituting a fourth epitope (Figures 5, 6). These results suggest that extra positive charges may facilitate the binding of PY-NLSs to the presumptive *P. sojae* Kapβ2. Similar combinatorial organization of a PY-NLS was observed in the *Xenopus* Kapβ2 substrate ELYS (RRTRRRIIAKPVTRRKMR), in which a variant PY dipeptide (PV) is flanked by a C-terminal basic cluster (Lau et al., 2009). Thus, our results may explain why the four PY-NLS prototypes from human and yeast functioned poorly in *P. sojae*, as they lack the additional basic residues required for efficient targeting to the *P. sojae* nucleus.

### For nuclear import of highly conserved ribosomal and histone proteins, *P. sojae* requires combinations of NLSs that are autonomous in other eukaryotes

Like the nuclear entry mediated by the *P. sojae* cNLS and PY-NLS motifs, entry by highly conserved nuclear proteins also showed distinctive features in *P. sojae*. Amino acid sequences of many ribosomal proteins and core histones are highly conserved among different eukaryotes, though within an organism different conserved proteins are imported into the nucleus via different NLSs. This suggests that the responsible karyopherins from different organisms should show similar specificity. However, in *P. sojae*, NLS motifs that could act autonomously in human and yeast were required to act together to deliver conserved proteins into the nucleus. For example, in yeast L28, two sequences (7-KTRKHRG-13 and 24-KHRKHPG-30) could individually serve as an NLS. However, the corresponding sequences in *P. sojae* L28 were required together for efficient nuclear localization (thus constituting a bipartite NLS). In the case of histone H3, where the amino acid sequences are nearly identical in all eukaryotes, two adjacent sequences that could each serve as an NLS in human (residues 1–32 and 33–75 of H3) were jointly required for NLS activity in *P. sojae*. Similarly, in H4 three adjacent sequences that could serve separately as NLSs in human were required together in *P. sojae* H4 for nuclear entry. The results suggest that the *P. sojae* karyopherins that presumably bind to these NLSs do so more weakly than in other characterized eukaryotes, and perhaps therefore that binding by multiple karyopherins is required to produce nuclear entry in *P. sojae*.

### Software for prediction of functional NLS regions in oomycete proteins

During the course of this study, we evaluated three software tools for predicting regions of the proteins that might have NLS activity, conferring nuclear localization. *PSORT II* (PSORT, 1997) was released in 1997, and since then, a variety of experimentally defined NLSs have been found that do not match any of the consensus sequences (Kosugi et al., 2009). Based on those findings, additional NLS prediction tools have been developed to include those newly identified NLS. We evaluated *NLStradamus* and *cNLS Mapper*. We also evaluated *PredictNLS*, but it produced so few predictions as to not be useful, as has been reported elsewhere (Nguyen Ba et al., 2009). Overall we found that *NLStradamus* and *cNLS Mapper* identified long regions but with little indication of which amino acid residues might be most responsible for nuclear localization (Tables S3, S4). On the other hand, *PSORT II* identified large numbers of very specific predicted NLSs; this was useful at the outset of our study when we were searching for sequences important for nuclear localization in *P. sojae*, though in the end many of these proved to be false positives (in *P. sojae*). After we had experimentally tested a large number of protein segments for nuclear localization activity in *P. sojae*, it emerged that *NLStradamus* was most successful in identifying regions that could confer partial or full nuclear localization in *P. sojae*, albeit only 60% successful (Tables S3, S4). Of 10 regions predicted in 9 *P. sojae* proteins, six were wholly or partially correct, while four were incorrect. Of the four, two were false positives and two were false negatives. More accurate prediction tools for oomycetes will need to take into account the combinatorial nature of NLSs we observed in *P. sojae*, as exemplified most strikingly by PHYSO_251824.

In summary, using *P. sojae* as a model, we identified distinctive features of nuclear localization in oomycetes, based on characterization of multiple classes of NLS from 10 nuclear proteins. A consistent pattern has emerged in which individual NLS sequences that are sufficient for autonomous nuclear localization in other eukaryotes function weakly or not at all in *P. sojae*, but can collaborate with each other or with patches rich in basic residues to produce efficient localization. Several studies have shown that karyopherins from different families and organisms exhibit preferences for specific NLSs (Sekimoto et al., 1997; Köhler et al., 1999; Fang et al., 2001; Mason et al., 2002; Kosugi et al., 2009; Chang et al., 2012); further characterization of *P. sojae* karyopherin/NLS structures may help us to explain the distinctive features that we observed in our studies. Because nuclear localization has not been dissected comprehensively in a wide diversity of eukaryotes or even in other oomycetes, it is currently unclear whether the differences we observe in *P. sojae* are part of a wider pattern of diversity in eukaryotes, or have a narrower functional significance, related perhaps to the pathogenic lifestyle of this organism.

## Author contributions

YF and BT conceived the study. YF performed 90% of the experiments. HJ and GW performed human cell transfections; DW contributed technical assistance; YF wrote the manuscript with help of BT. GW also helped to edit the manuscript. All authors reviewed the manuscript and are accountable for its accuracy.

### Conflict of interest statement

The authors declare that the research was conducted in the absence of any commercial or financial relationships that could be construed as a potential conflict of interest.

## Supplementary material

Figure S1 **Artifacts caused by DAPI (4′, 6-diamidino-2-phenylindole) staining for live-cell imaging of *P. sojae* hyphae**. To label the *P. sojae* nuclei, *P. sojae* transformants expressing candidate NLSs fused to 2XGFP were stained with DAPI (final concentration, 0.2 μg/ml) for 25 min before imaging. **(A)** Different subcellular localizations of the NLS-tagged GFPs in *P. sojae* transformants with and without DAPI staining. Arrows indicate residual GFP staining of nuclei after DAPI treatment. **(B)** A representative image showing various localization patterns of 2XGFP fused to a synthetic NLS, PsNLS (Fang and Tyler, 2016). Region (a): GFP reporter showing nuclear localization in a small region of DAPI-stained hyphae. Region (b): Cytoplasmic localization in a larger region of DAPI-stained hyphae; arrowheads indicate artifactual cytoplasmic localization of NLS-GFP fusions. Region (c): Exclusive nuclear accumulation typically found in DAPI-free hyphae. **(C)** Time-lapse experiment tracking the process of mis-localization of PsNLS-2XGFP upon DAPI treatment. Red rectangles highlight changes occurring during DAPI incubation. In this example, the nuclear-localized PsNLS-2XGFP was released into the cytoplasm following 18–18.5 min incubation with DAPI. Nuclear disintegration occurs at different rates in different regions of the hyphae possibly because newer hyphal regions absorb DAPI more slowly. Also see Supplemental Video 1.

Figure S2 **Detailed mutational analysis of the PY-NLS candidate PHYSO_561151 reveals that an extended bipartite cNLS at the C-terminus is actually responsible for its nuclear localization. (A)** Domain structure of PHYSO_561151. Position of the candidate PY-NLS sequence (non-functional) within PHYSO_561151 is indicated by a black rectangle; the shortest bipartite cNLS responsible for the nuclear import of the protein is marked by a gray rectangle. The corresponding amino acid sequences are listed below. Epitopes 1, 2, 3 of the putative PHYSO_561151 PY-NLS sequence are in bold and underlined. NLSs predicted by *PSORT II, NLStradamus*, and *NLS Mapper* are underlined by gray solid, dash, and dotted lines respectively. **(B)** Subcellular localization of PHYSO_561151 and mutants. Representative images are shown.

Figure S3 **Detailed mutational analysis of the PY-NLS candidate PHYSO_533817 reveals that residues 172–314 determine the nuclear accumulation. (A)** Domain structure of PHYSO_533817. Position of the candidate PY-NLS (non-functional) sequence within PHYSO_533817 is indicated by a black rectangle. The corresponding amino acid sequence is listed below. Epitopes 1, 2, 3 of the putative PY-NLS sequence are in bold and underlined. NLSs predicted by *PSORT II, NLStradamus*, and *NLS Mapper* are indicated by gray solid and dash lines respectively. **(B)** Subcellular localization of PHYSO_533817 and mutants. Representative images are shown.

Figure S4 **Sequences used for nuclear import of ribosomal proteins S22a and L3 in yeast do not show the same activities in *P. sojae*. (A,B)** Left panels, alignment of *P. sojae* ribosomal proteins S22a (PsS22a, PHYSO_287103) and L3 (PsL3, PHYSO_285779), with their orthologs in *Arabidopsi*s *thaliana* (At), human (Hs), and *Saccharomyces cerevisiae* (Sc), respectively. Asterisks on the top of each alignment indicate conserved residues among the S22a or L3 orthologs. Sequence highlighted in yellow, NLSs reported in yeast ribosomal proteins S22a (Timmers et al., 1999) and L3 (Moreland et al., 1985). No NLS sequences were predicted by *PSORT II, NLStradamus* or *cNLS Mapper* in PsS22a. In PsL3, NLSs predicted by *PSORT II* and *NLStradamus* are underlined by gray solid, dash lines respectively; no NLS sequences were predicted by *cNLS Mapper*. Right panels, various fragments of ribosomal proteins, were expressed as fusions to 2XGFP in *P. sojae* transformants and visualized by confocal microscopy. Representative images are shown.

Supplemental Video 1 **Mis-localization of PsNLS-2XGFP occurred in *P. sojae* hyphae during DAPI treatment**. Red rectangle highlights changes occurring during DAPI incubation.

Data Sheet 1 **Supplemental sequences of the five putative PY-NLS-containing proteins**.

Table S1 ***Phytophthora*** **protein identifiers in FungiDB and NCBI**.

Table S2 **Primers used in this study**.

Table S3 **Summary of conventional and predicted cNLS-containing fragments that were tested throughout this study**.

Table S4 **NLS predicted by different NLS predictors**.
