# Supplementary figures and images for: Distinctive Nuclear Localization Signals in the Oomycete Phytophthora sojae
Source: Front Microbiol. 2017 Feb 2;8:10. doi: 10.3389/fmicb.2017.00010 (PMC5288373; doi:10.3389/fmicb.2017.00010)

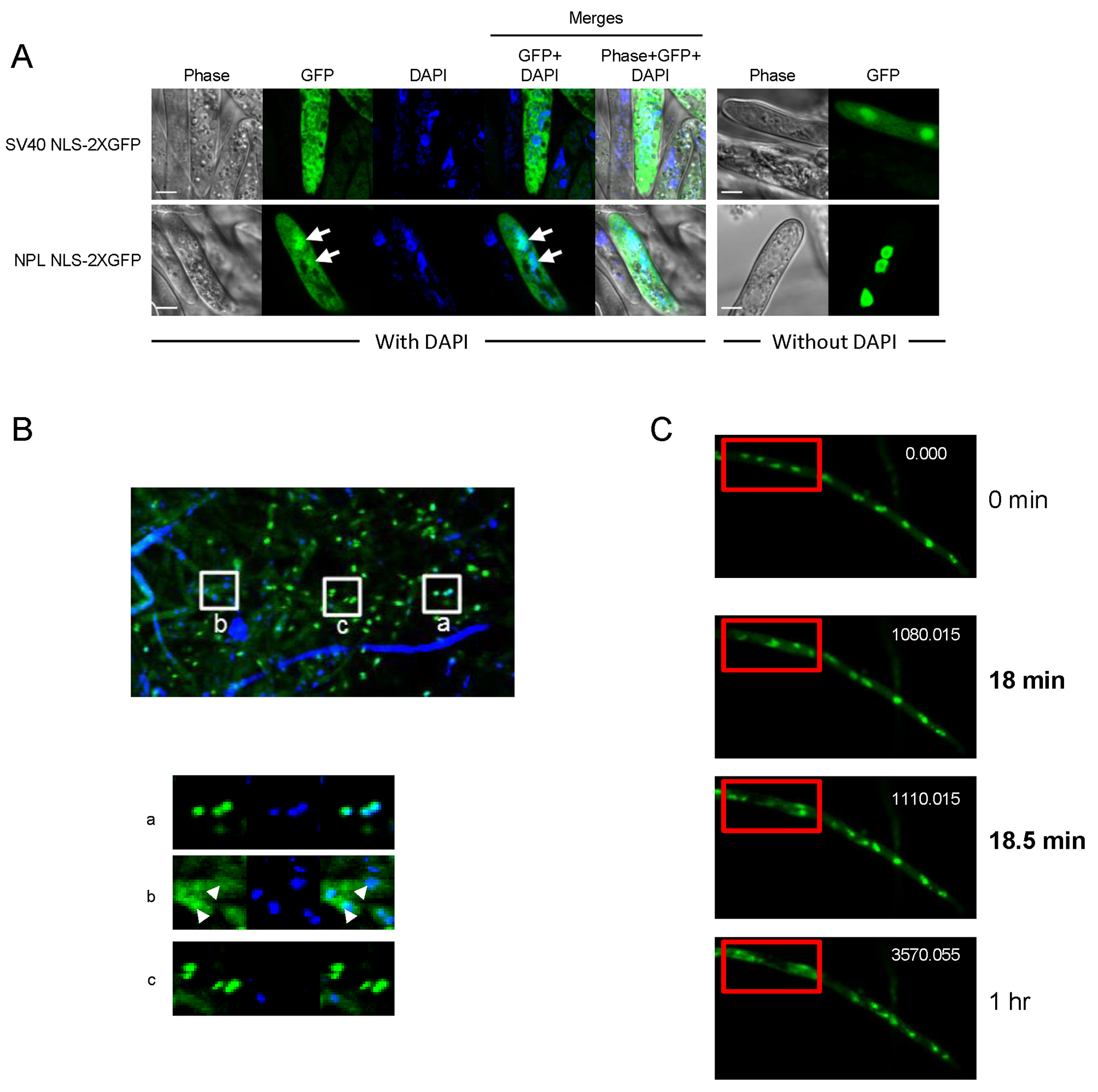

Supplement: Figure S1 — Artifacts caused by DAPI (4′, 6-diamidino-2-phenylindole) staining for live-cell imaging of P. sojae hyphae. To label the P. sojae nuclei, P. sojae transformants expressing candidate NLSs fused to 2XGFP were stained with DAPI (final concentration, 0.2 μg/ml) for 25 min before imaging. (A) Different subcellular localizations of the NLS-tagged GFPs in P. sojae transformants with and without DAPI staining. Arrows indicate residual GFP staining of nuclei after DAPI treatment. (B) A representative image showing various localization patterns of 2XGFP fused to a synthetic NLS, PsNLS (Fang and Tyler, 2016). Region (a): GFP reporter showing nuclear localization in a small region of DAPI-stained hyphae. Region (b): Cytoplasmic localization in a larger region of DAPI-stained hyphae; arrowheads indicate artifactual cytoplasmic localization of NLS-GFP fusions. Region (c): Exclusive nuclear accumulation typically found in DAPI-free hyphae. (C) Time-lapse experiment tracking the process of mis-localization of PsNLS-2XGFP upon DAPI treatment. Red rectangles highlight changes occurring during DAPI incubation. In this example, the nuclear-localized PsNLS-2XGFP was released into the cytoplasm following 18–18.5 min incubation with DAPI. Nuclear disintegration occurs at different rates in different regions of the hyphae possibly because newer hyphal regions absorb DAPI more slowly. Also see Supplemental Video 1. [file Image1.TIF]

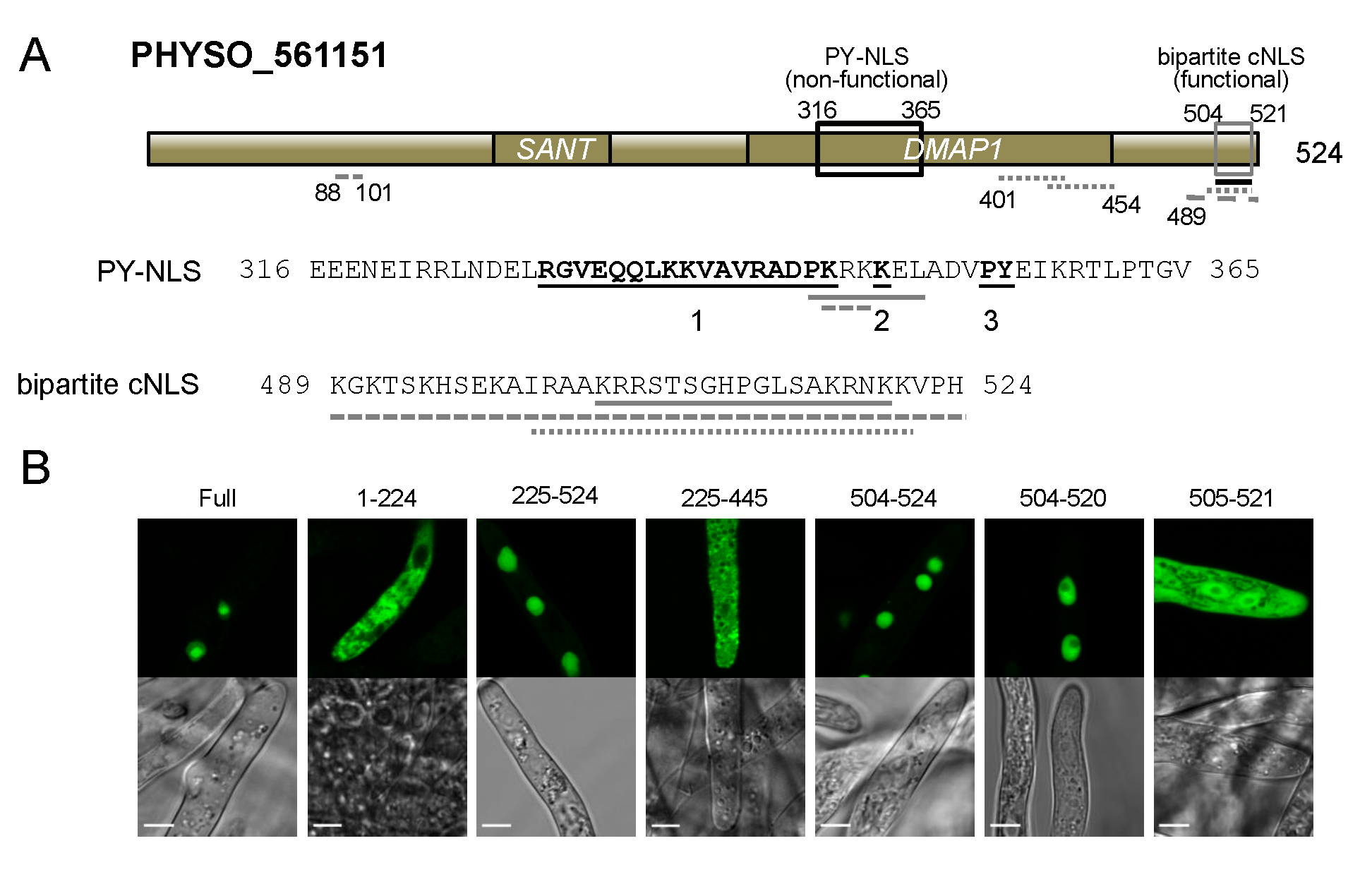

Supplement: Figure S2 — Detailed mutational analysis of the PY-NLS candidate PHYSO_561151 reveals that an extended bipartite cNLS at the C-terminus is actually responsible for its nuclear localization. (A) Domain structure of PHYSO_561151. Position of the candidate PY-NLS sequence (non-functional) within PHYSO_561151 is indicated by a black rectangle; the shortest bipartite cNLS responsible for the nuclear import of the protein is marked by a gray rectangle. The corresponding amino acid sequences are listed below. Epitopes 1, 2, 3 of the putative PHYSO_561151 PY-NLS sequence are in bold and underlined. NLSs predicted by PSORT II, NLStradamus, and NLS Mapper are underlined by gray solid, dash, and dotted lines respectively. (B) Subcellular localization of PHYSO_561151 and mutants. Representative images are shown. [file Image2.TIF]

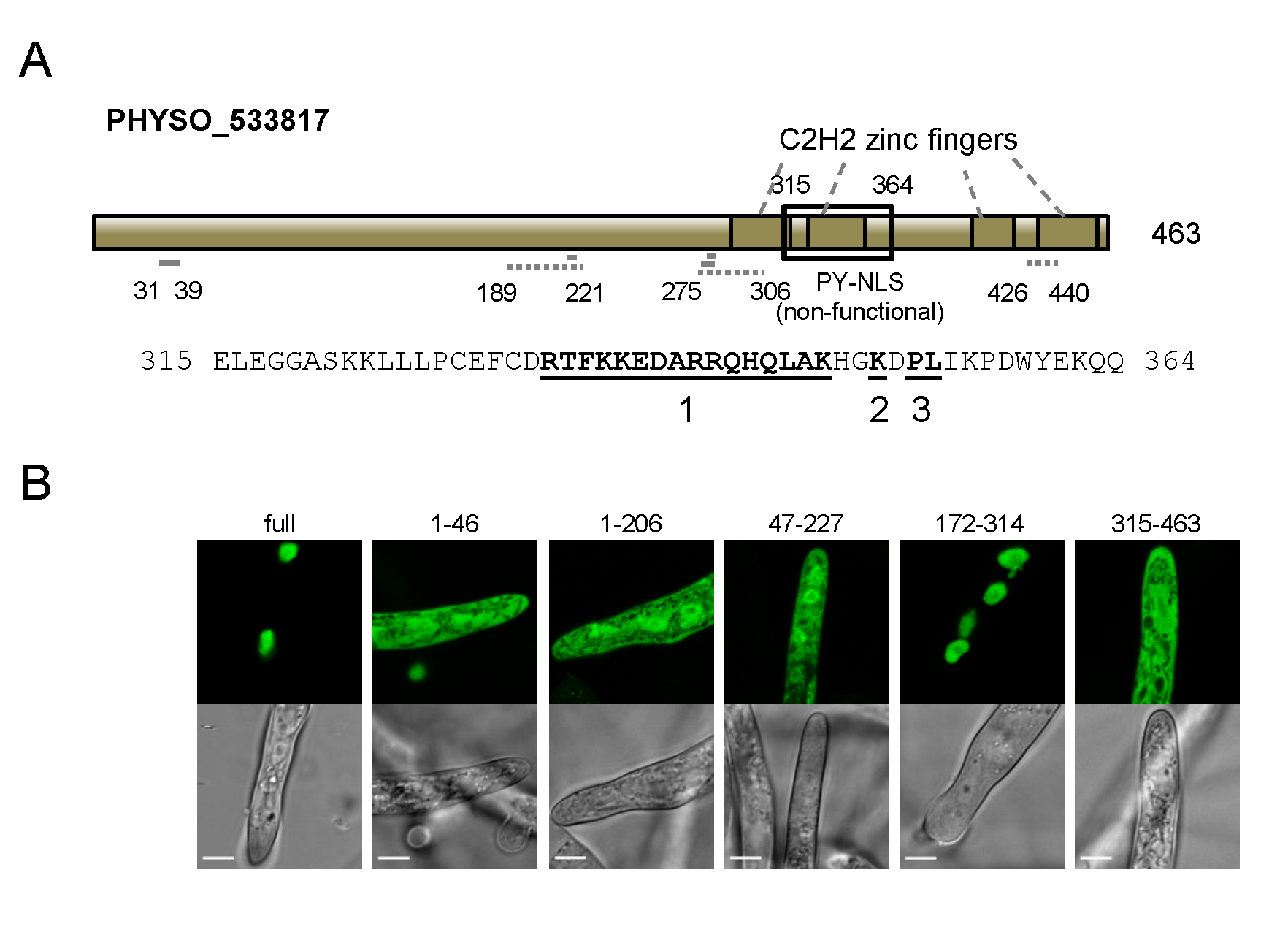

Supplement: Figure S3 — Detailed mutational analysis of the PY-NLS candidate PHYSO_533817 reveals that residues 172–314 determine the nuclear accumulation. (A) Domain structure of PHYSO_533817. Position of the candidate PY-NLS (non-functional) sequence within PHYSO_533817 is indicated by a black rectangle. The corresponding amino acid sequence is listed below. Epitopes 1, 2, 3 of the putative PY-NLS sequence are in bold and underlined. NLSs predicted by PSORT II, NLStradamus, and NLS Mapper are indicated by gray solid and dash lines respectively. (B) Subcellular localization of PHYSO_533817 and mutants. Representative images are shown. [file Image3.TIF]

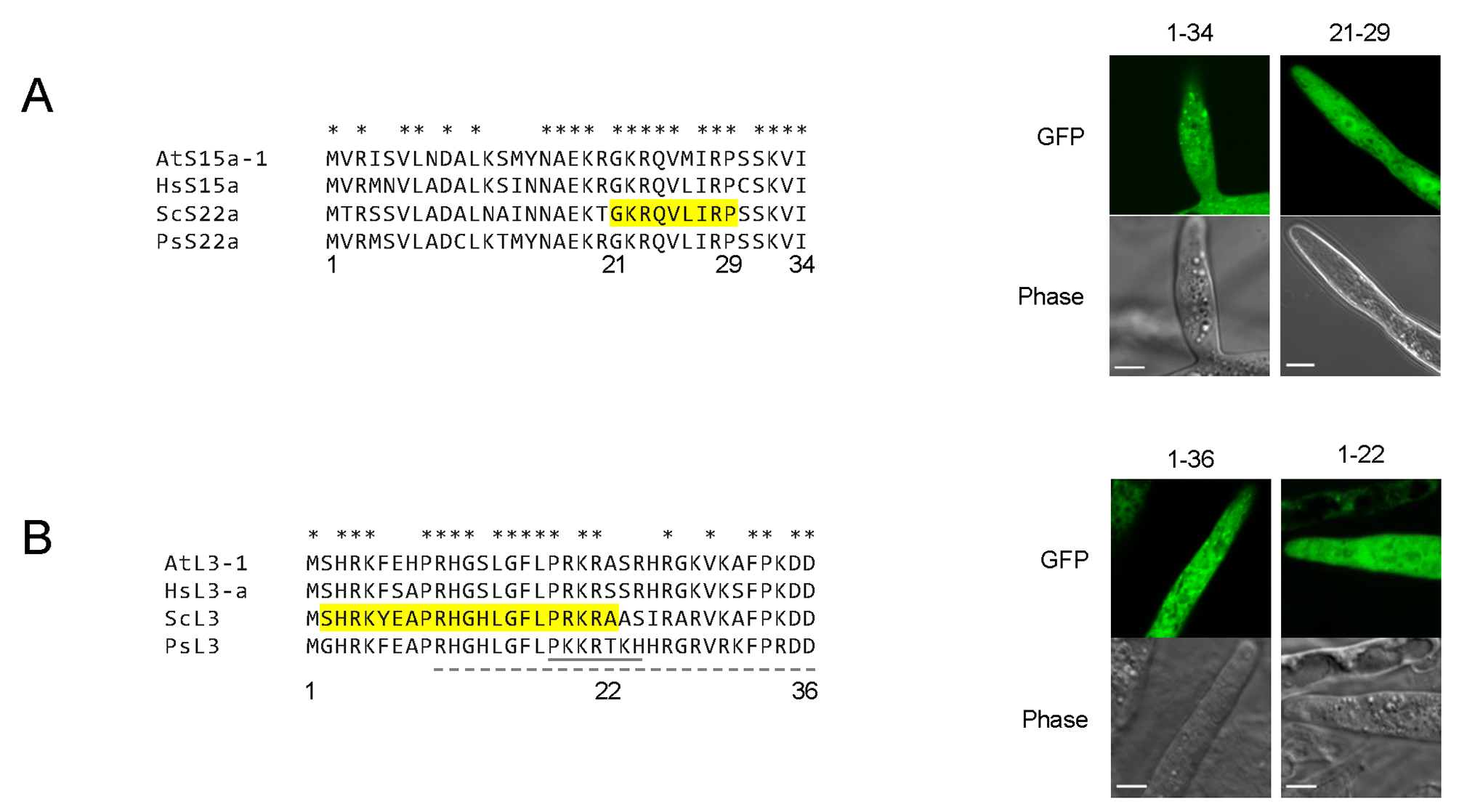

Supplement: Figure S4 — Sequences used for nuclear import of ribosomal proteins S22a and L3 in yeast do not show the same activities in P. sojae. (A,B) Left panels, alignment of P. sojae ribosomal proteins S22a (PsS22a, PHYSO_287103) and L3 (PsL3, PHYSO_285779), with their orthologs in Arabidopsis thaliana (At), human (Hs), and Saccharomyces cerevisiae (Sc), respectively. Asterisks on the top of each alignment indicate conserved residues among the S22a or L3 orthologs. Sequence highlighted in yellow, NLSs reported in yeast ribosomal proteins S22a (Timmers et al., 1999) and L3 (Moreland et al., 1985). No NLS sequences were predicted by PSORT II, NLStradamus or cNLS Mapper in PsS22a. In PsL3, NLSs predicted by PSORT II and NLStradamus are underlined by gray solid, dash lines respectively; no NLS sequences were predicted by cNLS Mapper. Right panels, various fragments of ribosomal proteins, were expressed as fusions to 2XGFP in P. sojae transformants and visualized by confocal microscopy. Representative images are shown. [file Image4.tif]
