# Supplementary material for: Distinctive Nuclear Localization Signals in the Oomycete Phytophthora sojae
Source: Front Microbiol. 2017 Feb 2;8:10. doi: 10.3389/fmicb.2017.00010 (PMC5288373; doi:10.3389/fmicb.2017.00010)
Supplement: Data Sheet 1 — Supplemental sequences of the five putative PY-NLS-containing proteins. [file DataSheet1.DOCX]

**Supplemental sequences of the five putative PY-NLS-containing proteins**

Notes:

Amino acids in lowercase and italics: incorrect intron annotation

Amino acids highlighted in yellow: sequences of candidate basic PY-NLS

Amino acids highlighted in green: sequences of candidate hydrophobic PY-NLS

Central basic or hydrophobic motifs (epitope I) are underlined and the R/K/H-PY motifs are in red bold

Amino acids in bold and italics: putative cNLS predicted by *PSORTII*, or largest region covering multiple overlapping cNLSs.

The basic patch proximal to the core PY-NLS-like sequence in PHYSO_480605 is highlighted in dark.

> PHYSO_357835|U3 small nucleolar RNA-associated protein

MATSGEFKRLVLKQFPATTEVETAENTYWKKFHAPQELQQVGPVTHIDVSPVAPHQVAITSSTRIHLYSTTTNEIVKTFSRFRDVVYSGTFRSDGKLLVAGGEAPYVQVLDINTRAILRSFKGHSAAIRSTRFSADNVHVLSCSDDKTSRYWDLPTGKPLALLGEHSDYVRSSAANPSSHNVWATGSYDHTVKLWDLRASDQTVSKSTMSLDHGAPVESCMIMPGGSLLLSAGGNSIKVWDILSGGRLLHSFSSHQKTITSLGLDGSGTRLMSGSLDGHLKIYDLKTYELAHGFKYKSGVLAFGMSPSNSHLFAGTVDGILAVRRRTVKRAEQTDAKNRQAIIRGGSYKYFLRGKNAQPAPADYTVATT***RHKR***IQ**PY**DRALRKFDYKKALNEALDTRSPVVVASMLEELRLRVGLKRALGGRDEETLEPLLAFLIKYVTDPKYASLLIHVCTIVCDLYAPKLSQSMLIDSLFVKLREKLNEELRVQKQVLGVVGMMDSVMAAQSNGTIGVDASS

>PHYSO_480605|mRNA cleavage and polyadenylation factor II complex, BRR5 (CPSF subunit)

MSKRRLAEEAADER**H**IMRIM**PL**GAGNEVGRSCIVLKFKGKTIMLDCGVHPGYSGHGSLPFFDGVEAEEIDLLLITHFHIDHVAALPHFTEKTNFKGRVFMTHPTKAVMQMMLRDFLRVSNISVDDQIYDDKDLNNCVSKVEIIDFHQEIMHNGIKFTPYNAGHVLGACMYLIEIGGVKVLYTGDYSLENDRHLMAAELPACSPDVLIVESTYGVQVHQSVVEREGRFTGQVEAVVRRGGRCLIPVFALGRTQELLLILDEHWRSHPDLQDIPIYFASKLAAKALRVYQTYINMMNDRIRKQIAISNPFQFEHISNLKSMDDFDDSGPSVVMASPGMLQSGVSRQLFERWCSDKRNACLIPGYVVEGTLAKKILSEPTEIAALDGRIIPMNCTVEYISFSAHADFVGTSGFVEKLTPPNIVLVHGEKNEMMRLKSALNKKFNDPKVYHPSISTPANMQEIVLEFKGEKIAKAIGGLASDQPKNGKVISGLLVEVDSQTHLMDKEDLSTYTKLISGSITQKQHVPFEYNSFDVLITFIRQMYEDVVHLETENRVVVCKQVVVTRCPVAKGATEKLVVEWTSAPTADMIADSVIALAMHAQASPASFKLSGQPTAACPHDHSKKEDESAHNEGAATKTEEEETP*vmetediaenppsehdaekke*AESDLEKAARELGEADQDALNLLIVFRLLKDQYGDVDLDFETNKIHVRTPSGIDAVVDHALQEIECKDAAFKLKLQTTVRRIEGALKPIATS

>PHYSO_251824|mRNA cleavage and polyadenylation factor I complex, subunit RNA15

MSNKSRASSAKERSVFVGNIPYDVTEDMLKEIFSEAGSVVNFRLVTDRETGKPKGYGFCEYADGATALSAMRNLNGYEINGRNLRVDFADGGDKSGGAD***RKRH***DNGSHARHGGTSGSNFRNGGSDGGPPTMVTGEMAIHAIESAIARLGPVKLYDMLVQLKEHARQKPEVTKSILMANPALTHAIVQSFKTLQIPIPSSTETQPVLLAPPPMMHQPPLMAGRMMPPPPRPLMGGHMGPGPGILGMAPPGVQPGMMNPAPAPAPAKSGGTRWSA**R**PG**PL**AAQATSSAMGGRPAVGIPASSPMHAQPQPGLMPTPTGPPQSSGPPSSNLAHASRDPRRAGRDPRLA**K**R**PY**PGEQGLAPPTGDND***PSKRSKP***SGFGADAAPGGQFDAIAELARDMTPQKLDMLPPNERQMLLAFMQQNNIPF

>PHYSO_561151|DNA methyltransferase 1-associated protein-1

MSDVAQILGLAGPKSGANGAAASELDQLKPTGASPAVRGKQSGGASKQKKLTGMQREVLELLESNHRASHALYQGFGKTTLKQKWQERKKSPAVKWLRKSFRNPARAGLPGESGEEGLVLTHWGKAHVEQPDYVFARFNVKCDTTSYTDEEYEAALANHLDPMMKWTKEETDLLLKLCQRFDLRWVVVTDKYNSNPIAKSAPRSMEDIKYRYYEATRLLSEYRDKKTRGELEKKAA*vatpaatsagapategtpagaataatpaaatve*TGGATSTPSTPVLDTPASSTSEHYRFNIAYEKQRKRQLDLTFSRTAEEENEIRRLNDELRGVEQQLKKVAVRAD***PKRKKEL***ADV**PY**EIKRTLPTGVILRSSLLALPQQKHALSAKLLKKLQLFLDEMGVPARPMPTKPVCETFDKLRQDAVGLLSLRKHLKSKQNEVQALRERYHALTGKEYKPITTPVTLSERPGDAALPDGSSSAASAAGHAQSTISKGKTSKHSEKAIRAA**KRRSTSGHPGLSAKRNKK**VPH

> PHYSO_533817|C2H2 zinc finger protein

MTQQEADTVPTPPSDAAPSEAKAATPSIPS***PDPRRRRVL***TVGDGNFSYSLALAKQHKSKGDDTRPLQLTATSYDSYDELVAKYPESKRICAQLKELGASVLHRVDATNIRESLVAAGATSDKFHSVVFNHPHCGEENVRRHQSLLSHFYASALEVLEKGEGDEVEQSEESGILLTLAEGQPERWQAVQRALSAGLKLHRQVDNVDSDAKFGLEYE***RKRH***QNGKSFHQVTLHGERKKQASTLFIFRRQKAGEKVEAVEAEVAPVvdaaavttgdgerkS**RKRK**AESELPLEFACTQCERSFKSAQGLRTHVHMVHELEGGASKKLLLPCEFCDRTFKKEDARRQHQLAKHG**K**D**PL**IKPDWYEKQQAAAS*esaadagagdvakestas*APSATTEAPATAEPQTCSICQLSFATAQEFDAHWQKLQPRSAAKRKCATCSREFDEERALRQHQNFCSQSKPAGSSS
