# Supplementary material for: Distinctive Nuclear Localization Signals in the Oomycete Phytophthora sojae
Source: Front Microbiol. 2017 Feb 2;8:10. doi: 10.3389/fmicb.2017.00010 (PMC5288373; doi:10.3389/fmicb.2017.00010)
Supplement: Table S1 — Phytophthora protein identifiers in FungiDB and NCBI. [file Table1.docx]

**Table S1 | *Phytophthora* protein identifiers in FungiDB and NCBI**

| Name or annotation* | FungiDB ID | NCBI accession # | Notes | Latest Fungi DB ID |
| --- | --- | --- | --- | --- |
| Conserved WD40 repeat-containing protein | PHYSO_357835 | XP_009538606.1 |  | PHYSODRAFT_356172 |
| mRNA cleavage and polyadenylation factor II complex, BRR5 (CPSF subunit) | PHYSO_480605 | XP_009521134.1 | Incorrect intron annotation | PHYSODRAFT_478942 |
| mRNA cleavage and polyadenylation factor I complex, subunit RNA15 | PHYSO_251824 | XP_009537811.1 |  | PHYSODRAFT_251824 |
| DNA methyltransferase 1-associated protein-1 | PHYSO_561151 | XP_009528012.1 | Incorrect intron annotation | PHYSODRAFT_559488 |
| C2H2 zinc finger protein | PHYSO_533817 | XP_009538325.1 | Incorrect intron annotation | PHYSODRAFT_532154 |
| PsL28 | PHYSO_355737 | XP_009520762 |  | PHYSODRAFT_354074 |
| PsL3 | PHYSO_285779 | XP_009514874 |  | PHYSODRAFT_284116 |
| PsS22a | PHYSO_287103 | XP_009523228 |  | PHYSODRAFT_285440 |
| PsH2B | PHYSO_474922 | XP_009515672.1 |  | PHYSODRAFT_473259 |
| PsH3 | PHYSO_286415 | XP_009518876 |  | PHYSODRAFT_284752 |
| PsH4 | PHYSO_285922 | XP_009515675.1 |  | PHYSODRAFT_284259 |
| *P. capsici* fibrillarin (FIB) | PHYCA_506928 | KY452016 |  | N/A |

* The naming system for *P. sojae* ribosomal proteins follows the nomenclature of *S. cerevisiae*. All other protein annotations were adapted from FungiDB, except PHYSO_533817 which was annotated as a C2H2 transcription factor by fungal transcription factors database (FTFD, Park *et al*, 2008).

Additional references

Park, J., J. Park, S. Jang, S. Kim, S. Kong, J. Choi, K. Ahn, J. Kim, S. Lee & S. Kim, (2008) FTFD: an informatics pipeline supporting phylogenomic analysis of fungal transcription factors. *Bioinformatics* **24**: 1024-1025.
