# Supplementary material for: Distinctive Nuclear Localization Signals in the Oomycete Phytophthora sojae
Source: Front Microbiol. 2017 Feb 2;8:10. doi: 10.3389/fmicb.2017.00010 (PMC5288373; doi:10.3389/fmicb.2017.00010)
Supplement: Table S2 — Primers used in this study. [file Table2.docx]

**Table S2 | Primers used in this study.**

| Primer name | Sequence | Usage |
| --- | --- | --- |
| pYF2_GW_F | 5' GGGGACAAGTTTGTACAAAAAAGCAGGCTGCAGGAATTAATTCGATATCAAGCTTATC 3' | PCR amplify 2XGFP and its flanking restriction sites from pYF2 plasmid and fuse to pcDNA3.2 using Gateway® technology |
| GFP_stop_GW_R | 5' GGGGACCACTTTGTACAAGAAAGCTGGGTTCTACTTGTAGAGTTCATCCATGCCATG 3' |  |
| GFP_AflII_F | 5' CGG*CTTAAG*ATGGGCAAGGGCGAGGAAC 3' | Generate plasmid pYF3-2XGFP |
| GFP_CMCS_ApaI_R | 5' TAGGGCCCTCAACGCGTTCCGGAGTTAACGGATTCTGTACACTTGTAGAGTTCATCCATGCCATG 3' |  |
| SV40_NLS_SacII_F | 5' GGATGCCAAAGAAAAAGAGAAAGGTTA 3' | Oligo annealing, clone SV40 NLS to the N-terminus of 2XGFP |
| SV40_NLS_SpeI_R | 5' CTAGTAACCTTTCTCTTTTTCTTTGGCATCCGC 3' |  |
| SV40_NLS_BsrG1_F | 5' GTACACCAAAGAAAAAGAGAAAGGTTT 3' | Oligo annealing, clone SV40 NLS to the C-terminus of 2XGFP |
| SV40_NLS_BspEI_R | 5' CCGGAAACCTTTCTCTTTTTCTTTGGT 3' |  |
| 3XSV40_NLS_SacII_F | 5' GGATGCCAAAGAAAAAGAGAAAGGTTCCAAAGAAAAAGAGAAAGGTTCCAAAGAAAAAGAGAAAGGTTA 3' | Oligo annealing, clone three copies of SV40 NLS to the N-terminus of 2XGFP |
| 3XSV40_NLS_SpeI_R | 5' CTAGTAACCTTTCTCTTTTTCTTTGGAACCTTTCTCTTTTTCTTTGGAACCTTTCTCTTTTTCTTTGGCATCCGC 3' |  |
| c-Myc_NLS_SacII_F | 5' GGATGCCGGCCGCCAAGCGCGTGAAGCTGGACA 3' | Oligo annealing, clone c-Myc NLS to the N-terminus of 2XGFP |
| c-Myc_NLS_SpeI_R | 5' CTAGTGTCCAGCTTCACGCGCTTGGCGGCCGGCATCCGC 3' |  |
| NLP_NLS_SacII_F | 5' GGATGAAGCGCCCGGCCGCCACGAAGAAGGCCGGCCAGGCCAAGAAGAAGAAGA 3' | Oligo annealing, clone nucleoplasmin NLS to the N-terminus of 2XGFP |
| NLP_NLS_SpeI_RIV | 5' CTAGTCTTCTTCTTCTTGGCCTGGCCGGCCTTCTTCGTGGCGGCCGGGCGCTTCATCCGC 3' |  |
| PsH2B_PacI_F | 5' CC*TTAATTAA*ATGGCGAAGACTCCCTCG 3' | Clone *P. sojae* H2B to pGPFN or pMCherryN |
| PsH2B_NheI_R | 5' TT*GCTAGC*AGCGGACGTGAACTTGGT 3' |  |
| PsFIB_AgeI_F | 5' GGGACCGGTATGGCCGGTGGCGCCAAG 3' | Clone *P. capsici* fibrillarin to pMCherryN |
| PsFIB_PacI_R | 5' CGCC*TTAATTAA*CTTCTTCTCCTTCTTGGGCACACGG 3' |  |
| Ps561151_504_SacII_F | 5' GGATGAAGCGACGCAGCACCAGTGGCCACCCAGGTCTCTCTGCCAAGCGCAACAAGA 3' | Oligo annealing, clone  residues 504-520 of PHYSO_561151 to the N-terminus of 2XGFP |
| Ps561151_504_SpeI_R | 5' CTAGTCTTGTTGCGCTTGGCAGAGAGACCTGGGTGGCCACTGGTGCTGCGTCGCTTCATCCGC 3' |  |
| Ps561151_505_SacII_F | 5' GGATGCGACGCAGCACCAGTGGCCACCCAGGTCTCTCTGCCAAGCGCAACAAGAAAA 3' | Oligo annealing, clone  residues 505-521 of PHYSO_561151 to the N-terminus of 2XGFP |
| Ps561151_505_SpeI_R | 5' CTAGTTTTCTTGTTGCGCTTGGCAGAGAGACCTGGGTGGCCACTGGTGCTGCGTCGCATCCGC 3' |  |
| M9NLS_SacII_F | 5' GGATGTTCGGCAACTACAACAACCAGTCGTCGAACTTCGGCCCGATGAAGGGCGGCAACTTCGGCGGCCGCTCGTCGGGCCCGTACA 3' | Oligo annealing, clone  M9 NLS to the N-terminus of 2XGFP |
| M9NLS_SpeI_R | 5' CTAGTGTACGGGCCCGACGAGCGGCCGCCGAAGTTGCCGCCCTTCATCGGGCCGAAGTTCGACGACTGGTTGTTGTAGTTGCCGAACATCCGC 3' |  |
| hnRNPD_SacII_F | 5' GGATGTACGGCGACTACTCGAACCAGCAGTCGGGCTACGGCAAGGTGTCGCGCCGCGGCGGCCACCAGAACTCGTACAAGCCGTACA 3' | Oligo annealing, clone  hnRNPD NLS to the N-terminus of 2XGFP |
| hnRNPD_SpeI_R | 5' CTAGTGTACGGCTTGTACGAGTTCTGGTGGCCGCCGCGGCGCGACACCTTGCCGTAGCCCGACTGCTGGTTCGAGTAGTCGCCGTACATCCGC 3' |  |
| hnRNP-M_SacII_F | 5' GGATGGAGCGCCCGGCCCAGAACGAGAAGCGCAAGGAGAAGAACATCAAGCGCGGCGGCAACCGCTTCGAGCCGTACGCCAACCCGACGAAGCGCA 3' | Oligo annealing, clone  hnRNPM NLS to the N-terminus of 2XGFP |
| hnRNP-M_SpeI_R | 5' CTAGTGCGCTTCGTCGGGTTGGCGTACGGCTCGAAGCGGTTGCCGCCGCGCTTGATGTTCTTCTCCTTGCGCTTCTCGTTCTGGGCCGGGCGCTCCATCCGC 3' |  |
| M9M_NheI_F | 5' CGC*GCTAGC*ATGTTCGGCAACTACAACAACCAGTCG 3' | PCR amplify M9M using M9NLS as a template and clone it to the N-terminus of 2XGFP using *Stu I* of pYF2-2XGFP |
| M9M_AgeI_R | 5' GCC*ACCGGT*GCGCTTCGTCGGGTTGGCGTACGGCTCGAAGCGGCCGCCGAAGTTGCC 3' |  |
| Nab2p1_SacII_F | 5' GGATGGACAACTCGCAGCGCTTCACGCAGCGCGGCGGCGGCGCCGTGGGCAAGAACCGCCGCGGCGGCCGCGGCGGCAAC 3' | Oligo annealing, clone  Nab2p PY-NLS to the N-terminus of 2XGFP |
| Nab2p1_R | 5' GCCGCGGTTGCCGCCGCGGCCGCCGCGGCGGTTCTTGCCCACGGCGCCGCCGCCGCGCTGCGTGAAGCGCTGCGAGTTGTCCATCCGC 3' |  |
| Nab2p2_F | 5' CGCGGCGGCCGCAACAACAACTCGACGCGCTTCAACCCGCTGGCCAAGGCCCTGGGCATGGCCGGCGAGTCGAACATGA 3' |  |
| Nab2p2_SpeI_R | 5' CTAGTCATGTTCGACTCGCCGGCCATGCCCAGGGCCTTGGCCAGCGGGTTGAAGCGCGTCGAGTTGTTGTTGCGGCC 3' |  |
| Ps357835_F | 5' ACCATGGCGACCAGCGGCGAG 3' | PCR amplification of full length PHYSO_357835 |
| Ps357835_R | 5' ACTGCTGGCATCCACACCGATGGTG 3' |  |
| Ps357835_338_F | 5' ATGAACCGTCAGGCGATCATCCG 3' | PCR amplification of residues 338-378 in PHYSO_357835 |
| Ps357835_387_R | 5' CTTGTAATCAAACTTCCGCAGCG 3' |  |
| Ps357835_H371A/K372A | 5' ACGGCTGAATACGCGCGGCACGTGTGGTGGCCACGGTGT 3' | Mutate H371A/K372A in PHYSO_357835 |
| Ps357835_P376A/Y377A | 5' CGTCACAAGCGTATTCAGGCGGCCGACCGCGCGCTG 3' | Mutate P376A/Y377A in PHYSO_357835 |
| Ps357835_H371A/K372A/P376A/Y377A | 5' CAGCGCGCGGTCGGCCGCCTGAATACGCGCGGCACGTGTGGTGGCCAC 3' | Mutate H371A/K372A/P376A/Y377A in PHYSO_357835 |
| Ps357835(H371A/K372A)_R370A/R373A | 5' GTGGCCACCACAGCTGCCGCGGCTATTCAGGCGGC 3' | Further mutate R370A/R373A in PHYSO_357835(H371A/K372A) |
| Ps480605_F | 5' ATGTCGAAGCGCAGGCTAGCCG 3' | PCR amplify full length PHYSO_480605 and its truncations |
| Ps480605_R | 5' CGACGTAGCAATGGGCTTCAGTGC 3' |  |
| Ps480605_32_R | 5' CGCC*TTAATTAA*GCACGAGCGCCCCACCTCG 3' |  |
| Ps480605_60_R | 5' GAACGGCAGGCTGCCGTG 3' |  |
| Ps480605_33_F | 5' ATCGTGCTCAAGTTCAAGGGCAAGAC 3' |  |
| Ps480605_810_F | 5' CATCCCAATCTACTTTGCATCC 3' | Sequencing primer for PHYSO_480606 |
| Ps480605_1539_F | 5' GGGTAGTATCACCCAGAAGCA 3' | Sequencing primer for PHYSO_480607 |
| Ps251824_F | 5' ATGAGCAATAAGTCGCGCGCG 3' | PCR amplify full length PHYSO_251824 and its truncations |
| Ps251824_R | 5' AAACGGAATGTTGTTCTGCTGCATG 3' |  |
| Ps251824_238_R | 5' GCC*CCGCGG*TTAAAACGGAATGTTGTTCTGCTGCATG 3' |  |
| Ps251824_239_F | 5' ATGGGGCCAGGCATCCTCGGC 3' |  |
| Ps251824_309_F | 5' ATGTTGATGCCAACTCCGACTGGTC 3' |  |
| Ps251824_308_R | 5' GCCCGGTTGAGGCTGAGCG 3' |  |
| Ps251824_362_R | 5' GTCATTGTCTCCTGTCGGCGGTG 3' |  |
| Ps251824_cNLS2_F | 5' GGATGCCGTCGAAGCGCTCGAAGCCGA 3' | Oligo annealing, clone  residues 363-369 (the cNLS2 in PHYSO_251824) to the N-terminus of 2XGFP |
| Ps251824_cNLS2_R | 5' CTAGTCGGCTTCGAGCGCTTCGACGGCATCCGC 3' |  |
| Ps251824_cNLS_mut_R | 5' GGAGACAATGACCCGTCCGCGGCGTCCGCGCCGAGCGGTTTTGGTGC 3' | Mutate cNLS2 in residues 239-419 of PHYSO_251824 |
| Ps251824_P347A_Y348A | 5' CCCTCGTTTGGCAAAGAGAGCGGCTCCCGGCGAG 3' | Mutate P347A/348A in context of PHYSO_251824(P347A/348A) |
| Ps251824_R332A/R336A/R339A | 5' CACGCCAGCGCTGACCCTCGAGCTGCAGGCGCAGACCCTCG-3' | Mutate R332A/R336A/R339A in context of PHYSO_251824(P347A/348A) |
| Ps561151_F | 5' GCCTTAATTAAATGAGCGACGTGGCGCAGATCTTGG 3' | PCR amplify full length PHYSO_561151 and its truncations |
| Ps561151 _R | 5' ATGCGGGACTTTCTTGTTGC 3' |  |
| Ps561151 _225_F | 5' ATGAAGAAGACGAGAGGTGAACTTGAAA 3' |  |
| Ps561151 _445_R | 5' CGCGTGGTATCGTTCACGA 3' |  |
| Ps561151_cNLS504_SacII_F | 5' GGATGAAGCGACGCAGCACCAGTGGCCACCCAGGTCTCTCTGCCAAGCGCAACAAGA 3' | Oligo annealing, clone  residues 504-520 of PHYSO_561151 to the N-terminus of 2XGFP |
| Ps561151_cNLS504_SpeI_R | 5' CTAGTCTTGTTGCGCTTGGCAGAGAGACCTGGGTGGCCACTGGTGCTGCGTCGCTTCATCCGC 3' |  |
| Ps561151_cNLS505_SacII_F | 5' GGATGCGACGCAGCACCAGTGGCCACCCAGGTCTCTCTGCCAAGCGCAACAAGAAAA 3' | Oligo annealing, clone  residues 505-521 of PHYSO_561151 to the N-terminus of 2XGFP |
| Ps561151_cNLS505_SpeI_R | 5' CTAGTTTTCTTGTTGCGCTTGGCAGAGAGACCTGGGTGGCCACTGGTGCTGCGTCGCATCCGC 3' |  |
| Ps561151_del 225-403_R | 5' TGGTGCTGCGTCGCTTCATCCTATCGATAAGC 3' | Delete residues 225-403 in constructs pYF2-2XGFP-PHYSO_561151_225-524_ to generate residues 504-524 |
| Ps561151_del 225-403_F | 5' GCTTATCGATAGGATGAAGCGACGCAGCACCA 3' |  |
| Ps533817_F | 5' ATGACGCAGCAAGAAGCGGACAC 3' | PCR amplify full length PHYSO_ Ps533817 and its truncations |
| Ps533817_R | 5' ACTACTGCTACCGGCAGGCTTACTCTG 3' |  |
| Ps533817_46_R | 5' GAAATTCCCGTCGCCCACAGTG 3' |  |
| Ps533817_206_R | 5' GCTGTCCACGTTGTCAACCTGTCG 3' |  |
| Ps533817_47_F | 5' ATGTCGTACTCATTGGCGTTGGCCAAG 3' |  |
| Ps533817_227_R | 5' CTGGTGGAACGACTTGCCGTTCTG 3' |  |
| Ps533817_315_F | 5' ATGCACGAGTTGGAGGGGGGCG 3' |  |
| Ps533817_172_F | 5' ATGATCCTGTTGACGCTGGCCG 3' |  |
| Ps533817_314_R | 5' CACCATGTGCACGTGCGTCCTC 3' |  |
| Ps533817_315_F | 5' ATGCACGAGTTGGAGGGGGGCG 3' |  |
| PsL28_F | 5' TGCCGTCCCGTTTTTCCAAGAAC 3' | PCR amplify residues 1-33 of PsL28 |
| PsL28_R | 5' ACCGCGACCACCCGGGTG 3' |  |
| PsL28_NLS2-mus_R | 5' GTGCTAGCACCGGCACCACCCGGGTGCTT 3' | Mutate R7A/K9A/R10A in PsL28_1-33_ |
| PsL28_NLS1-mus_R | 5' CGCTGACGTGTCCGCGTTTCGCGGCGTTCGCGGAAAAACGGGACGGCATTTA-3' |  |
| PsL28-NLS1_SacII_F | 5' GGATGAACCGCAAGAAACGCGGAA 3' | Oligo annealing, clone NLS1 (residues 7-13) in PsL28 to N-terminus of 2XGFP |
| PsL28-NLS1_SpeI_R | 5' CTAGTTCCGCGTTTCTTGCGGTTCATCCGC 3' |  |
| PsL28-NLS2_SacII_F | 5' GGATGAAGCACCGCAAGCACCCGGGTA 3' | Oligo annealing, clone NLS2 (residues 24-30) in PsL28 to the N-terminus of 2XGFP |
| PsL28-NLS2_SpeI_R | 5' CTAGTACCCGGGTGCTTGCGGTGCTTCATCCGC 3' |  |
| PsS22A_F | 5' ATGGTTCGTATGAGCGTGCTGG 3' | PCR amplify residues 1-34 of PsS22A |
| PsS22A_34_R | 5' GATCACCTTGGAGCTCGGGC 3' |  |
| PsS22A_SacII_F | 5' GGATGGGAAAGCGCCAGGTGCTCATCCGCCCGA | Oligo annealing, clone residues 21-29 of PsS22A to the N-terminus of 2XGFP |
| PsS22A_SpeI _R | CTAGTCGGGCGGATGAGCACCTGGCGCTTTCCCATCCGC |  |
| PsL3_F | 5' ATGGGTCACCGTAAGTTCGAGGC 3' | PCR amplify residues 1-36 of PsL3 |
| PsL3_36_R | 5' GTCATCGCGCGGGAACTTGC 3' |  |
| PsL3_SacII_F | 5' GGATGGGTCACCGTAAGTTCGAGGCCCCGCGCCACGGCCACCTGGGTTTCCTGCCGAAGAAGCGCACCA 3' | Oligo annealing, clone residues 1-22 of PsL3 to the N-terminus of 2XGFP |
| PsL3_SpeI_R | 5' CTAGTGGTGCGCTTCTTCGGCAGGAAACCCAGGTGGCCGTGGCGCGGGGCCTCGAACTTACGGTGACCCATCCGC 3' |  |
| PsH3_F | 5' ATGGCCCGTACCAAGCAGACCG 3' | PCR amplify full length PsH3 and its truncations |
| PsH3_R | 5' CGAGCGCTCGCCACGGATG 3' |  |
| PsH3_32_R | 5' GGCGGGGGCCGACTTGC 3' |  |
| PsH3_33_F | 5' ATGACGGGCGGCGTCAAGAAAC 3' |  |
| PsH3_75_R | 5' GATCTCGCGCACCAGGCGC 3' |  |
| PsH3_76_F | 5' GCCCAGGACTTCAAGACGGAC 3' |  |
| PsH4_F | 5' ATGTCTGGACGCGGCAAAG 3' | PCR amplify full length PsH4 and its truncations |
| PsH4_R | 5' TCCGCCGAAGCCGTAGAG 3' |  |
| PsH4_25_R | 5' GTCGCGAAGAACCTTGC 3' |  |
| PsH4_42_R | 5' ACCACGGCGAGCGAGAC 3' |  |
| PsH4_80_R | 5' CTTGCGGCGCGCGTG 3' |  |
| PsH4_26_F | 5' ATGAACATCCAGGGCATCACCAAG 3' |  |
| PsH4_43_F | 5' ATGGGAGTGAAGCGCATCTCCG 3' |  |
| PsH4_81_F | 5' ATGACCGTGACGGCCATGGAC 3' |  |
