# Supplementary material for: Distinctive Nuclear Localization Signals in the Oomycete Phytophthora sojae
Source: Front Microbiol. 2017 Feb 2;8:10. doi: 10.3389/fmicb.2017.00010 (PMC5288373; doi:10.3389/fmicb.2017.00010)
Supplement: Table S3 — Summary of conventional and predicted cNLS-containing fragments that were tested throughout this study. [file Table3.pdf]

Table S3 | Summary of conventional and predicted cNLS-containing fragments that were tested throughout this study.

Name	Fragment containing predicted NLS[1]	pSORTII[2]	NLS-tradamus	cNLS Mapper	Nuc[5]
**SV40 cNLS**	PKKKRKV	-	-	-	I
c-myc	PAAKRVKLD	-	-	-	I
**NPL**	KRPAATKKAGQAKKKK	-	-	-	√
**PsL28**	1-MPSRFSKNRKKRGHVSAGHGRIGKHRKHPGGRG-33	M, B	Y[3]	Y	√
	8-NRKKRG-13	M	N[4]	N	×
	24-KHRKHPG-30	M	N	N	×
	11-KRGHVSAGHGRIGKHRK-27	B	N	N	×
**PsL3**	1-MGHRKFEAPRHGHLGFLPKKRTKHHRGRVRKFPRDD-36	M	Y	N	×
**PsH3**	76-...116KRVTIMPKDIQLARRIRGERS-136	B	N	N	×
**PsH4**	1-MSGRGKGGKGLGKGGAKRHRKVLRD-25	M	N	N	×
**PHYSO_251824**	1-...100RKRH103...-238	M	N	N	×
	363-PSKRSKP-369	M	N	Y	×
**PHYSO_561151**	225-...344PKRKKEL350...-455	M	Y	N	×
	504-KRRSTSGHPGLSAKRNK-520	B	Y	Y	√
	505-RRSTSGHPGLSAKRNKK-521	B	Y	Y	×
**PHYSO_533817**	1-...PDPRRRRVLTVGDGNF-46	M	N	N	×

1. Sequences predicted by *PSORT II* as NLSs in large fragments are underlined. Some sequences may have multiple cNLSs. Due to the limited space, long flanking sequences are abbreviated (indicated by dots).

2. cNLS patterns classified by *PSORT II*: M, monopartite; B, bipartite.

3. Y, the fragment shown overlaps with a putative NLS predicted by *NLStradamus* or *cNLS Mapper*.

4. N, the fragment shown does not overlap with a putative NLS predicted by *NLStradamus* or *cNLS Mapper*.

5. √ = the NLS-containing sequence showed clear nuclear targeting activity. I, incomplete nuclear localization (GFP signal is visible in the cytoplasm, approximately 1<LNC<3), × = little nuclear localization.
