# Supplementary material for: Distinctive Nuclear Localization Signals in the Oomycete Phytophthora sojae
Source: Front Microbiol. 2017 Feb 2;8:10. doi: 10.3389/fmicb.2017.00010 (PMC5288373; doi:10.3389/fmicb.2017.00010)
Supplement: Table S4 — NLS predicted by different NLS predictors. [file Table4.pdf]

Table S4 | NLS predicted by different NLS predictors.

Proteins tested in our study	Residues showed nuclear targeting activity[1]	Predicted NLS		
		pSORT II[2]	NLStradamus[3]	cNLS mapper[4]
PsL28$_{1-33}$	1-33	pat4, 9-RKKR-12<br>pat4, 24-KHRK-27<br>bipartite,<br>11-KRGHVSAGHGRIGKHRK-27	2s, 6-SKNRKKRGHVSAGHGRIGKHRKHPGGRG-33<br>2d,4s, 7-KNRKKRGHVSAGHGRIGKHRKHPGGRG-33	bipartite,<br>4-RFSKNRKKRGHVSAGHGRIGKHRKHP-29<br>(11.5)
PsS22a$_{1-34}$	None	None	None	None
PsS22a	N/A	pat4, 117-RRKH-120	None	None
PsL3$_{1-36}$	None	pat4, 18-PKKR-21<br>pat7, 18-PKKRTKH-24	2s,2d, 18-PKKRTKHHRGRVRKFPRDD - 36<br>2d, 14-LGFLPKKRTKHHRGRVRKFPR - 34<br>4s, 10-RHGHLGFLPKKRTKHHRGRVRKFPR-34	None
PsH3	1-75	bipartite,<br>116-KRVTIMPKDIQLARRIR-132	2s, 8-ARKSTGGKAPRKQLATKAARKSAPATGGVKKPHRYRP-44<br>2d, 7-TARKSTGGKAPRKQLATKAARKSAPATGGVKKPHRYRP-44<br>4s, 9-RKSTGGKAPRKQLATKAARKSAPATGGVKKPHRYR-43	None
PsH4	1-42 (I)<br>1-80<br>26-80 (I)	pat4, 17-KRHR-20<br>pat4, 18-RHRK-21	2s,<br>4-RGKGGKGLGKGGAKRHRKVLRDNIQGITKPAIRRLARRGGVKR-46<br>2d/4s,<br>4-RGKGGKGLGKGGAKRHRKVLRDNIQGITKPAIRRLARRGGVK-45	None
PHYSO_357835	338-387	370-RHKR-373	No	6-EFKRLVLKQFPATTEVETAENTYWKKFHAP-35 (5.9)<br>410-RLRVGLKRALGGRDEETLEPLLAFLIKYVTDP-441 (6.9)
PHYSO_480605	1-32 (I)<br>1-60	None	None	None

Table S4, continued

Proteins tested in our study	Residues showed nuclear targeting activity	Predicted NLS		
		pSORT II	NLStradamus	cNLS mapper
PHYSO_251824	239-419	100-RKRH-103<br>363-PSKRSKP-369	2s,2d, No<br>4s, 335-RRAGRDPRLAKR-346	bipartite,<br>339–<br>RDPRLAKRPYPGEQGLAPPTGDNDPSKRSKPS-370 (7.7)<br>339-<br>RDPRLAKRPYPGEQGLAPPTGDNDPSKRSKP-369 (5.0)<br>339-<br>RDPRLAKRPYPGEQGLAPPTGDNDPSKRSKPSG-371 (6.0)
PHYSO_561151	504-520	pat4, 344-PKRK-347<br>pat4, 345-KRKK-348<br>pat7, 344-PKRKKEL-350<br>bipartite,<br>504-KRRSTSGHPGLSAKRNK-520<br>bipartite,<br>505-RRSTSGHPGLSAKRNKK-521	2s,<br>88-RKKSPAVKWLRKSF-101<br>490-GKTSKHSEKAIRAAKRRSTSGHPGLSAKRNKKVPH-524<br>2d,<br>88-RKKSPAVKWLRK-99<br>489-KGKTSKHSEKAIRAAKRRSTSGHPGLSAKRNKKVPH-524<br>4s,<br>88-RKKSPAVKWLRK-99<br>345-KRK-347<br>491-KTSKHSEKAIRAAKRRSTSGHPGLSAKRNKKVP-523	bipartite,<br>401-<br>RPMPTKPVCETFDKLRQDAVGLLSLRKHLKSK-432 (6.6)<br>425-<br>LRKHLKSKQNEVQALRERYHALTGKEYKPI-454 (6.3)<br>500-IRAAKRRSTSGHPGLSAKRNKK-521 (6.9)

Table S4, continued

Proteins tested in our study	Residues showed nuclear targeting activity	Predicted NLS		
		pSORT II	NLStradamus	cNLS mapper
PHYSO_533817	172-314	pat4, 33-PRRR-36<br>pat4, 34-RRRR-37<br>pat4, 216-RKRH-219<br>pat4, 279-RKRK-282<br>pat7, 31-PDPRRRR-37<br>pat7, 33-PRRRRVL-39	2s,2d, 278-SRKR-281<br>4d, 277-KSRKRK-282	monopartite,<br>276-RKSRKRKAESE-286 (14.0)<br>426-RSAAKRKCATCSREF-440 (5.0)<br>426-RSAAKRKCATC-436 (6.0)<br>bipartite,<br>189-RALSAGLKLHRQVDNVDSDAKFGLEYERKRHQN-221 (6.4)<br>275-ERKSRKRKAESELPLEFACTQCERSFKSAQG-305 (8.7)<br>275-ERKSRKRKAESELPLEFACTQCERSFKSAQGL-306 (5.3)

[1.] I, incomplete nuclear localization (GFP signal is visible in the cytoplasm, approximately 1<LNC<3).

[2.] *PSORTII* differentiates monpartite and bipartite cNLSs. The detection and classification of monopartite cNLSs is based on two rules: pat 4, 4 residue pattern, which composes of 4 basic amino acids (K or R), or composed of three basic amino acids (K or R) and either H or P. pat 7, 7 residue pattern, which start with P and followed within 3 residues by a basic segment containing 3 K/R residues out of 4. Ref. PSORT, (1997).

[3.] NLStradamus does not classify NLS types. The NLS prediction is based on three hidden markov models (HHM). 2s, 2 state HHM static; 2d, 2 state HHM dynamic; 4s, 4 state HHM static. Ref. Ba et al, (2009).

[4.] *cNLS Mapper* generates and distinguishes monopartite and bipartite cNLSs. The predictions shown are based on a cut-off score, 5.0 (a candidate protein fused reporter localizes to both the nucleus and the cytoplasm). Score for each predicted NLS is shown in a parenthesis at the end of the sequence. Ref. Kosugi et al., (2009).
